# Morph-SSL: Self-Supervision With Longitudinal Morphing for Forecasting AMD Progression From OCT Volumes

**DOI:** 10.1109/TMI.2024.3390940

**Published:** 2024-09-03

**Authors:** Arunava Chakravarty, Taha Emre, Oliver Leingang, Sophie Riedl, Julia Mai, Hendrik P.N. Scholl, Sobha Sivaprasad, Daniel Rueckert, Andrew Lotery, Ursula Schmidt-Erfurth, Hrvoje Bogunović

**Affiliations:** Department of Ophthalmology and Optometry, Medical University of Vienna, 1090 Vienna, Austria; Department of Ophthalmology and Optometry, Medical University of Vienna, 1090 Vienna, Austria; Department of Ophthalmology and Optometry, Medical University of Vienna, 1090 Vienna, Austria; Department of Ophthalmology and Optometry, Medical University of Vienna, 1090 Vienna, Austria; Department of Ophthalmology and Optometry, Medical University of Vienna, 1090 Vienna, Austria; Institute of Molecular and Clinical Ophthalmology Basel, 4031 Basel, Switzerland, and also with the Department of Ophthalmology, University of Basel, 4001 Basel, Switzerland; NIHR Moorfields Biomedical Research Centre, Moorfields Eye Hospital NHS Foundation Trust, EC1V 2PD London, U.K.; BioMedIA, Imperial College London, SW7 2AZ London, U.K.; Institute for AI and Informatics in Medicine, Klinikum rechts der Isar, Technical University of Munich, 80333 Munich, Germany; Clinical and Experimental Sciences, Faculty of Medicine, University of Southampton, SO17 1BJ Southampton, U.K.; Department of Ophthalmology and Optometry, Medical University of Vienna, 1090 Vienna, Austria; Department of Ophthalmology and Optometry and the Christian Doppler Laboratory for Artificial Intelligence in Retina, Medical University of Vienna, 1090 Vienna, Austria

**Keywords:** Self-supervised learning, disease progression, age-related macular degeneration, retina, longitudinal OCT

## Abstract

The lack of reliable biomarkers makes predicting the conversion from intermediate to neovascular age-related macular degeneration (iAMD, nAMD) a challenging task. We develop a Deep Learning (DL) model to predict the future risk of conversion of an eye from iAMD to nAMD from its current OCT scan. Although eye clinics generate vast amounts of longitudinal OCT scans to monitor AMD progression, only a small subset can be manually labeled for supervised DL. To address this issue, we propose Morph-SSL, a novel Self-supervised Learning (SSL) method for longitudinal data. It uses pairs of unlabelled OCT scans from different visits and involves morphing the scan from the previous visit to the next. The Decoder predicts the transformation for morphing and ensures a smooth feature manifold that can generate intermediate scans between visits through linear interpolation. Next, the Morph-SSL trained features are input to a Classifier which is trained in a supervised manner to model the cumulative probability distribution of the time to conversion with a sigmoidal function. Morph-SSL was trained on unlabelled scans of 399 eyes (3570 visits). The Classifier was evaluated with a five-fold cross-validation on 2418 scans from 343 eyes with clinical labels of the conversion date. The Morph-SSL features achieved an AUC of 0.779 in predicting the conversion to nAMD within the next 6 months, outperforming the same network when trained end-to-end from scratch or pre-trained with popular SSL methods. Automated prediction of the future risk of nAMD onset can enable timely treatment and individualized AMD management.

## I Introduction

**A**GE-RELATED macular degeneration (AMD) is a leading cause of blindness in the elderly population [1]. Although asymptomatic in its early and intermediate stages, it gradually progresses to a late stage leading to irreversible vision loss. Early or intermediate AMD (iAMD) is primarily characterized by the presence of drusen. Additionally, the Retinal Pigment Epithelium (RPE) and Photoreceptor (PR) layers degenerate over time and are associated with Hyper-reflective Foci (HRF). The late stage is characterized by significant vision loss either due to the presence of Geographic Atrophy (GA) called dry AMD, the presence of choroidal neovascularisation (CNV) called neovascular AMD (nAMD), or a combination of both. nAMD is caused by the abnormal growth of blood vessels that leak fluid into the retina [2] which can be effectively treated with intravitreal anti-VEGF injections. If patients at a higher risk of conversion to nAMD can be identified in the iAMD stage itself, then potential future vision loss could be avoided through frequent monitoring and early treatment. However, the rate of progression varies widely across patients. There are no reliable biomarkers in the iAMD stage to differentiate between slow and fast progressors making it difficult for clinicians to determine the precise risk and timing of conversion. Thus, deep learning (DL)-based methods to predict the future risk of conversion to nAMD can play a critical role in enabling patient-specific disease management.

Optical Coherence Tomography (OCT) provides a 3D view of the retinal tissue and comprises a series of cross-sectional 2D image slices called B-scans. In clinical practice, a longitudinal series of OCT scans is routinely acquired over multiple patient visits to assess and monitor AMD progression. It generates a large amount of retrospective imaging data that can potentially be used to train DL models. However, due to the time, effort, and clinical expertise required, manual Ground Truth (GT) labels are rarely available for supervised training. Self-Supervised Learning (SSL) offers a way to address this issue by training DL networks to solve *pretext* tasks on unlabelled training data to learn useful feature representations.

In this work, we propose a novel SSL method specifically adapted to longitudinal datasets called Morph-SSL. It involves morphing an OCT scan from one visit to a future visit scan of the same eye. We surmise that the change between the features extracted from two visits should reflect the structural deformation and the intensity changes between them. Morph-SSL is employed to develop a prognostic model to predict future conversion from iAMD to nAMD within the next *t* months from a single current OCT scan. *t* can be any continuous time-point up to a maximum of 18 months. We refer to this task as TTC, predicting the probability distribution of the *Time-to-Conversion*. Once an Encoder has been trained with Morph-SSL, a 3-layer Classifier is trained for the TTC task on limited labelled data. The Encoder and Classifier can further be fine-tuned jointly. Our key contributions are:

(i) We propose Morph-SSL, a novel SSL method to learn representations that capture temporal changes in the retinal tissue from unlabelled longitudinal datasets. With minimal constraints on the unlabelled training data, Morph-SSL requires at least two visits per eye and can also use scans acquired at irregular intervals. The learned feature manifold is enforced to be smooth with meaningful notions of distance and direction, such that linear interpolation between two scans in the feature space leads to a gradual transition between them in the image space.
(ii) We model the Cumulative Distribution Function (CDF) of the probability of the TTC with a sigmoidal function over time. It allows using continuous GT labels of conversion time during training, ensures the monotonic non-decreasing property of the CDF, and can predict the conversion risk for arbitrary continuous time-points at test time.
(iii) We propose a score *r* ∈ [0, 1] that quantifies the future risk of eyes to develope nAMD and can categorize them into low and high risk groups for conversion. *r* can play a crucial role in personalized treatment by identifying the high risk patients for early treatment and more frequent monitoring.
(iv) We develop an efficient CNN network to process entire OCT volumes instead of individual 2D B-scans. We explore (a) *S3DConv* block to replace 3D convolutions with three groups of 2D convolutions oriented in the three orthogonal planes; (b) concatenation-based (instead of additive) skip connections to have the same output channel size with fewer convolutions; (c) Layer Normalization instead of Batch Normalization to allow training with a batch size of 1.

## II Related Work

### A Self-Supervised Learning

It offers a way to overcome the paucity of labelled datasets for supervised training. SSL learns feature representations from unlabelled data by training the network on a pretext task that does not need manual labels. SSL-trained models can either be utilized for off-the-shelf feature extraction or to provide initial weights for fine-tuning on the desired *downstream* task with limited labelled training data. Recent SSL methods employ pretext tasks based on image reconstruction or Contrastive Learning (CL). Reconstruction-based methods train networks to predict the original image from its distorted version and have been applied to X-ray, CT, MRI and ultrasound images [3], [4]. The distortions involve transformations such as non-linear intensity mapping, local shuffling, and in-painting in Model-Genesis [3] and randomly swapping patches in the image [4].

CL has been applied to chest X-ray, dermatology [5], histology [6], MRI [7] and ultrasound [8] images. CL trains networks using random batches comprising two data-augmented versions per image, called *positive pairs*. While positive pairs are pulled closer, the features of different images in the batch called *negative pairs* are pushed apart. However, the images in a negative pair can still be semantically similar (same pathology or disease stage), resulting in many *False Negative pairs*. Their impact can be reduced by training with large batch sizes (1024 for chest X-rays, 512 for dermatology images in [5] and 128 for histology image patches in [6]). Since large batch sizes do not scale well to 3D images due to limited GPU memory, existing methods learn features at a 2D, slice-level for 3D MRI volumes [7], or for individual frames in ultrasound videos [8] where neighboring slices/frames of the same 3D image are excluded from negative pairs. The recently proposed *Non-Contrastive* methods overcome the problem of *False Negative pairs*. They do not maximize the negative pair separation but only ensure that they do not collapse onto the same feature representation. VICReg [9] keeps the standard deviation of each feature dimension over a batch above a threshold. Barlow Twins [10] forces the cross-correlation between two batch of features extracted from the two images in each positive pair to be close to the identity matrix. BYOL [11] prevents feature collapse using slightly different network weights to extract features for the two views in the positive pair, where the second network weight is computed as the moving average of past weights.

CL and Non-Contrastive SSL have been adapted for retinal OCT to learn features for 2D B-scans with training batch sizes of 128 in [12] and 384 in [13]. Another method learns features for central B-scans by predicting the time interval between two input scans from random visits of the same patient [14]. In contrast, *Morph-SSL with a novel image morphing-based pretext task can be trained with a batch size of 1 to reduce GPU memory usage, allowing us to learn feature representations for entire 3D OCT volumes instead of 2D B-scans*.

### B Time to Conversion Prediction

Existing methods either employ Color Fundus Photographs (CFP) or OCT imaging for TTC prediction. CFP is a 2D image of the retinal surface and lacks a cross-sectional view of the retina. A 9-grade AREDS disease severity scale [15] further stratifies the iAMD stage in CFPs, where each successive stage has been linked to an increased 5-year risk of conversion to advanced AMD (from 1% in grade 1 to about 50% risk in grade 9). However, no such severity scale exists for the relatively new OCT imaging. The establishment of imaging biomarkers in the iAMD stage preceding the onset of nAMD remains a topic of ongoing investigation, as evidenced by many recent [2], [16], [17], [18] and ongoing clinical studies [19]. In [16], morphological changes such as hyperreflective foci (HRF) and pigment epithelial detachment (PED) were observed one month before the manifestation of choroidal neovascularization (CNV). Additionally, an increase in drusen area or thickness [2], [17], HRF [17] and the presence of a thick Double-Layer Sign (characterized by the visible separation of the retinal pigment epithelium and Bruch’s Membrane with different reflectivity) [18] have emerged as potential biomarkers indicative of an elevated risk of converting to nAMD within a two-year time frame. Moreover, an accelerated thinning and appearance changes in the choroid was also observed in [20] prior to nAMD onset within a one year follow-up period.

Some *CFP-based methods* predict the AREDS severity scale [21], [22]. Two-year conversion of nAMD was predicted with an ensemble of such predictions combined with features from drusen segmentation and demographic data [22]. The CNN-LSTM based methods in [23] and [24] require images from multiple past visits, hence cannot be used for patients visiting for the first time. The input CFPs from visits at irregular intervals are handled by scaling the input image features with visit time intervals [23] or using a time-aware LSTM network [24]. In [25], a Generative Adversarial Network was used to generate synthetic CFP images for future time-points. Combining CFP with genetic features can improve performance [26], but such information is not readily available in eye clinics. While CFP-based methods can predict long-term conversion, they are not sensitive to short-term conversion risks within 2 years, required for effective clinical intervention. Because CFPs lack a 3D view of the retina, they cannot capture subtle changes in retinal layers or extract accurate lesion volumes.

Many *OCT-based methods* first extract a set of handcrafted quantitative biomarkers to capture the distribution, appearance and volume of lesions like drusen, HRF and retinal layers such as RPE and PR. These biomarkers combined with other demographic [27] or genetic data [28] are input to an LSTM [27], Cox proportional hazards model [28], or an L1-penalized Poisson model [29] to predict the TTC. The biomarkers are extracted with automated segmentation methods that are often inaccurate and require voxel-level labels to train. Moreover, handcrafted biomarkers may not adequately capture the subtle retinal changes related to disease progression.

Another approach directly uses the OCT scans as input. To reduce the compute and GPU memory, most existing methods operate on individual B-scans with 2D CNNs. Some methods in [14] and [12] only use the central B-scan that passes through the macula, ignoring the remaining B-scans in the volume. While [14] employed this strategy for TTC prediction, [12] used it on other tasks such as predicting age, sex and visual acuity from OCT. Alternatively, 2D CNNs can be applied independently to every B-scan in the volume. During inference, the predictions from each B-scan is pooled, either by taking the average [30] or maximum [13] to obtain the volume-level prediction. During training, the same GT for the conversion time is used for every B-scan in the volume, even if only a few of them have the biomarkers indicative of progression risk, resulting in noisy training labels. *In contrast to these methods, in this work we explore a full 3D approach by developing a compact 3D-CNN network to effectively capture the spatial information across the individual B-scans*. Other than our work, the only other 3D-CNN method is found in [31], which employs two prediction networks to predict the conversion risk within six months, one using raw OCT volumes and the other using retinal layer and lesion segmentation maps.

## III Method

Our primary contribution is Morph-SSL, a new Self-Supervised Learning method designed to leverage the wider availability of unlabeled longitudinal training data. It learns feature representations that are sensitive to the morphological characteristics in an input OCT scan which are indicative of future disease progression. Morph-SSL uses pairs of unlabeled scans acquired at irregular (but known) time-intervals from each subject to solve the *pretext* task of morphing the scan from the prior visit to the next as detailed in III-A. Considering implementation challenges such as GPU memory and the computation time required in a 3D convolutional network, we also developed a lightweight CNN architecture (in Fig. 2) for processing 3D OCT volumes.

In order to demonstrate the effectiveness of our learned representations, we chose a clinically relevant downstream task of predicting the future risk of conversion of eyes (currently in the iAMD stage) to the late nAMD stage in Section III-B. The Cumultative Distribution Function (CDF) of the time-to-conversion (TTC) is modeled as a sigmoid function over time. The CDF parameters are predicted using a classifier that employs the Morph-SSL trained encoder.

**Fig. 1:**
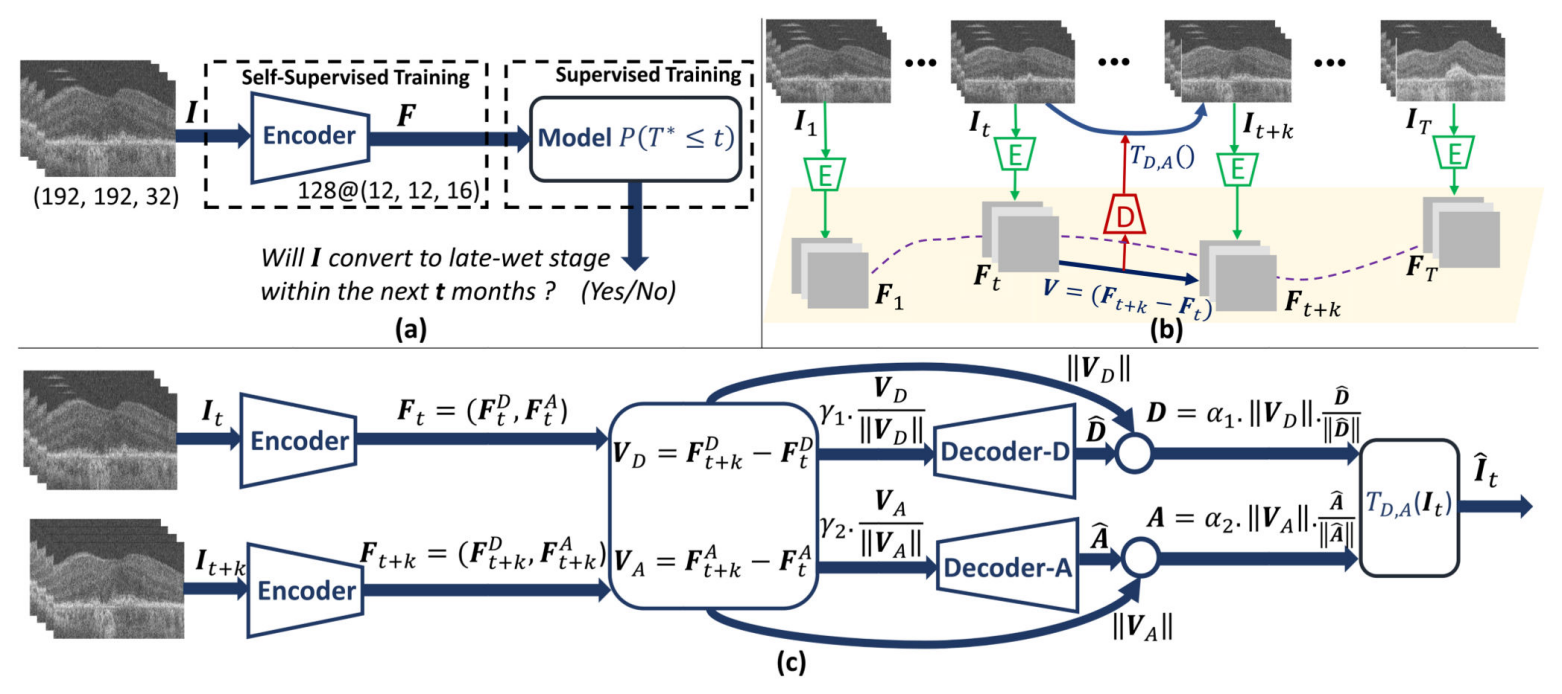
**(a)** Overview of our 2-stage training framework: Stage-1 involves self-supervised pre-training of an Encoder using Morph-SSL. Stage-2 involves supervised training of a classifier for the downstream task of predicting future nAMD conversion within the next *t* months. Optionally, an end-to-end fine-tuning of the pre-trained Encoder and the classifier can be performed. The motivation behind Morph-SSL is shown in **(b)** and its details in **(c)**. Please refer to Fig. 2(c) for the Encoder and Fig. 3(b) for the classifier architecture.

The overall pipeline combining the *unsupervised* representation learning and the *supervised* downstream conversion prediction stage is depicted in Fig. 1(a). First, a Fully Convolutional Encoder is trained with Morph-SSL to project an input OCT scan **I** to a convolutional feature map **F**. This stage only utilizes unlabeled longitudinal image-pairs for training. Next, **F** is input to a classifier for the downstream task of predicting the future conversion to nAMD within the next *t* months. This task involves modeling the CDF of conversion time using a sigmoid distribution whose parameters are predicted by the classifier. By freezing the Morph-SSL pre-trained weights for the encoder, the classifier is trained in a supervised manner on limited labeled data. Optionally, an additional end-to-end finetuning can also be performed using the Morph-SSL pre-trained weights for network initialization.

### A Self-Supervised Learning

#### 1) Motivation

Let {**I**_*t*_ |1 ≤ *t* ≤ *T*} represent a set of 3D OCT scans of an eye acquired over *T* visits, in which **I**_*t*_ is obtained on the *t*^*th*^ visit. The Encoder projects each **I**_**t**_ to a feature map **F**_*t*_ of size 128@12×12×16. **F**_*t*_ can be interpreted as 128-dimensional features for overlapping 3D image patches in **I**_*t*_, with the patch size defined by the effective receptive field of the Encoder. As AMD progresses over successive visits, **F**_*t*_ traces a trajectory (denoted by the violet dotted line in Fig. 1(b)) that is locally linear between nearby visits **I**_*t*_, and **I**_*t*+*k*_ (assuming a smooth feature manifold) but maybe non-linear over the entire AMD progression. Let 𝒯_*D,A*_(.) denote a transformation which morphs **I**_*t*_ to look similar to **I**_*t*+*k*_, with parameters **D** and **A**. As **I**_*t*_ morphs into **I**_*t*+*k*_ in the image space, **F**_*t*_ should be linearly displaced to **F**_*t*+*k*_ by **V**_*t*_ = **F**_*t*+*k*_ − **F**_*t*_ in the feature manifold. This observation motivates our pretext task for Morph-SSL which employs an Encoder-Decoder architecture. The Encoder projects scans from two nearby visits, **I**_*t*_ and **I**_*t*+*k*_ to their features **F**_*t*_ and **F**_*t*+*k*_. The Decoder uses the displacement **V**_*t*_ as input to predict **D** and **A** of the morphing transformation 𝒯_*D,A*_. Our pretext task ensures that the displacements in the learned feature manifold capture the corresponding appearance changes in the image space.

𝒯_*D,A*_ comprises a spatial deformation with the 3-channel **D** and an additive intensity transformation with the 1-channel **A**, both of the same spatial size as **I**_*t*_. Each voxel at location ***p*** in **I**_*t*_ is displaced to the location ***p*** + **D**(***p***), where **D**(***p***) is a 3-dimensional displacement vector representing the translations along the height, width and depth direction. Additionally, **A**(***p***) captures the intensity changes at each location ***p***, caused by newly formed pathologies in **I**_*t*+*k*_ such as fluids or drusen. Thus, the transformed image is ${\hat{\text{I}}}_{t}={𝒯}_{D,A}({\text{I}}_{t})=\text{Φ}({\text{I}}_{t};\text{D})+\text{A}$, where Φ is the spatial deformation applied in a differentiable manner similar to the registration methods in [32] and [33] based on the Spatial Transformer Networks [34]. **A** has a single color channel (similar to **I**_*t*_) to model the additive intensity transformation since OCTs are grayscale images.

#### 2) Morph-SSL Framework

The details are depicted in Fig. 1(c). **F**_**t**_ is split into two subspaces ${\text{F}}_{t}^{D}$ and ${\text{F}}_{t}^{A}$ of 64 channels each. The Decoder has two sub-networks, *Decoder-D* and *Decoder-A* that operate on ${\text{F}}_{t}^{D}$ and ${\text{F}}_{t}^{A}$ feature maps respectively, to predict **D** and **A**. A notion of semantically meaningful directions and distance is incorporated. The amount of deformation between **I**_*t*_ and **I**_*t*+*k*_ should be proportional to the Euclidean distance $||V_{D}||=∥F_{t+k}^{D}-F_{t}^{D}{∥}_{2}$, while the nature and location of the deformation should be captured by the direction alone, represented by the unit vector ***V***_*D*_/||***V***_*D*_||. This property is enforced by our Decoder architecture in Fig. 1(c). Only the direction information *γ*_1_.(***V***_*D*_/||***V***_*D*_||) is input to *Decoder-D* and its output $\hat{\text{D}}$ is normalized and scaled to obtain $\text{D}=\alpha_{1}\cdot ∥V_{D}∥\cdot (\hat{\text{D}}/∥\hat{\text{D}}∥)$. This ensures that ||**D**|| = *α*_1_.||**V**_*D*_||. Both *γ*_1_, *α*_1_ are learnable parameters (positive scalar weights) employed for numerical stability during training. A similar scheme is employed to predict **A**. The direction *γ*_2_.(***V***_*A*_/||***V***_*A*_||) is input to *Decoder-A* and its output scaled to $\text{A}=\alpha_{2}\cdot ∥V_{A}∥\cdot (\hat{\text{A}}/∥\hat{\text{A}}∥)$, where *γ*_2_, *α*_2_ are learnable positive weights (see Fig. 1(c)).

##### a) Loss function

The Encoder-Decoder network is trained to minimize the Mean Squared Error (MSE) between ${\hat{\text{I}}}_{t}$ and **I**_*t*+*k*_ by directly comparing their voxel intensities (ℒ_*mse*_) as well as their feature maps extracted with a CNN (ℒ_*prc*_). ℒ_*mse*_ alone leads to blurred reconstructions which is remedied by using the additional *perceptual loss* ℒ_*prc*_ [35], [36]. The OCT scans have a dark noisy background region both above and below the retinal tissue. We define the region between the Inner Limiting Membrane (ILM) and the Bruchs Membrane (BM) along with a small margin below the BM (to include the choroid) as the region of interest (ROI) containing the retinal tissue. The binary ROI mask **R**_*t*_ for the scan **I**_*t*_ is extracted automatically (see Section IV, *Preprocessing* section for details). While registering **I**_*t*_ to **I**_*t*+*k*_, we aim to morph the ROI in **I**_*t*_ to the corresponding retinal tissue region in **I**_*t*+*k*_. The background noisy region is ignored while computing ℒ_*mse*_ and ℒ_*prc*_ using **R**_*t*_ (for **I**_*t*_) and **R**_*t*+*k*_ (for **I**_*t*+*k*_), obtained during pre-processing. Before computing the loss, the background regions are masked out through element-wise multiplication **I**_*t*+*k*_ = **I**_*t*+*k*_ ⊙ **R**_*t*+*k*_ and ${\hat{\text{I}}}_{t}={\hat{\text{I}}}_{t}⊙\text{Φ}({\text{R}}_{t};\text{D})$. The Encoder does not require the binary masks at inference time as they are only used to compute the loss.

ℒ_*mse*_ has two terms. First, the MSE is computed with the only spatially deformed image Φ(**I**_*t*_ ; **D**). Next, **A** is fitted to the residual difference left after the spatial deformation, **U** = **I**_*t*+*k*_ − Φ(**I**_*t*_ ; **D**).*detach*(). The *detach*() indicates that the gradients are not allowed to backpropagate through **U** which is computed on the fly and treated as the GT for **A**. This two-step design is to ensure that **D** accounts for most of the reconstruction and avoid trivial solutions where **D** is an identity transformation (0 displacement for all voxels) while **A** tries to learn the entire difference **I**_*t*+*k*_ − **I**_*t*_. Thus,

(1) $${ℒ}_{mse}=\frac{\lambda_{1}}{\left|\text{Ω}\right|}\cdot ∥{\text{I}}_{t+k}-\text{Φ}({\text{I}}_{t};\text{D}){∥}_{2}^{2}+\frac{\lambda_{2}}{\left|\text{Ω}\right|}\cdot ∥\text{U}-\text{A}{∥}_{2}^{2},$$

where |Ω| is the total number of voxels in the image and the relative weights *λ*_1_ = 10^1^, *λ*_2_ = 10^2^ were set empirically.

The MSE in the voxel intensity space encourages smoothed reconstructions, blurring the edges and textural content in ${\hat{\text{I}}}_{t}$. A perceptual loss term ℒ_*prc*_ addresses this issue by extracting convolutional feature maps for **I**_*t*+*k*_ and ${\hat{\text{I}}}_{t}$ with a *Comparator* CNN network *ψ* and computing the MSE in the extracted feature space (rather than the raw voxel intensities) as

(2) $${ℒ}_{prc}=\frac{1}{3}\sum_{j=1}^{3}\frac{1}{\left|\text{Ω}\right|}∥\psi_{j}({\text{I}}_{t+k})-\psi_{j}({\hat{\text{I}}}_{t}){∥}_{2}^{2}.$$

*ψ*_*j*_ (**I**) denotes the feature map from the *j*^*th*^ layer of *ψ* for the input **I**. Typically, the first few layers of a pre-trained network such as VGG-16 are used for *ψ* [35], [36]. In the absence of a suitable pre-trained 3D CNN network for OCT volumes, we define *ψ* to have an architecture identical to the first 3 layers of our Encoder. Inspired by BYOL [11], *ψ* maintains a separate copy of its network weights which is updated with an exponential moving average of the past Encoder weights (for the first 3 layers) as the training proceeds. Although initially *ψ* is randomly initialized, the quality of its feature maps improves gradually during training. Thus, we eliminate the need for an existing pre-trained network for *ψ*.

Additional *regularization* loss terms are also incorporated to obtain an anatomically feasible 𝒯_*D,A*_. **D** is encouraged to be diffeomorphic by penalizing it to be smooth with ℒ_*smth*_ and prevent folding with ℒ_*fld*_. The ${ℒ}_{\text{smth}}={\text{∑}}_{p\in \text{Ω}}∥\nabla \text{D}(p){∥}_{2}^{2}$ was defined as in [32], where the spatial gradient ∇**D**(**p**) is computed at all voxel positions through discrete numerical approximation. ℒ_*f* *ld*_ as defined in [33], penalizes the anatomically infeasible deformations where the retinal tissue folds onto itself. Finally, the sparsity of **A** is ensured with an L1-regularization ℒ_*add*_ =∑ _*p*∈Ω_ |**A**(***p***)|. Thus, the total loss is

(3) $$ℒ={ℒ}_{mse}+\lambda_{3}\cdot {ℒ}_{prc}+\lambda_{4}\cdot {ℒ}_{smth}+\lambda_{5}\cdot {ℒ}_{fld}+\lambda_{6}{ℒ}_{add},$$

where λ_3_ = 10^1^, λ_4_ = 10^−1^, λ_5_ = 10^6^ and λ_6_ = 10^−5^ are empirically fixed, based on their relative importance and also to scale the different loss terms to a similar range. The range of the *ℒ*_*f* *ld*_ is orders of magnitude lower than the other terms, thus requiring a significantly larger scaling weight.

#### 3) Network Architecture

**Fig. 2:**
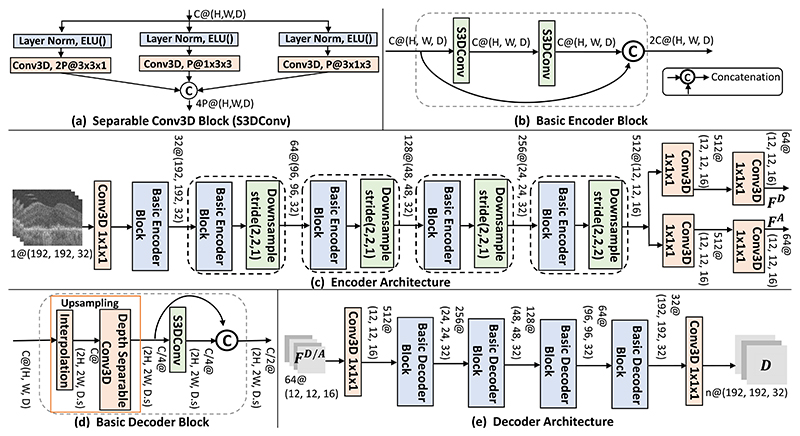
The S3DConv block in (a) is used as the basic convolution operation in our entire Encoder and Decoder architecture. A series of Basic Encoder Blocks detailed in **(b)** constitutes our Encoder architecture as shown in **(c)**. The Decoder-D and Decoder-A accept **F**^*D*^ and **F**^*A*^ as input respectively and employ a similar architecture comprising a series of Basic Decoder Blocks in **(d)**, except the number of output channels in the last layer (*n* = 3 for Decoder-D and *n* = 1 for Decoder-A) as depicted in **(e)**.

The *separable 3D Convolution Block* (S3DConv) depicted in Fig. 2(a) replaces 3D convolutions throughout our Encoder and Decoder Networks to reduce computation and network parameters. It employs 2D convolution filters in the three orthogonal planes. While 50% of the filters are 3×3×1 that operate on individual B-scans, the remaining are an equal number of 1×3×3 and 3×1×3 filters to capture contextual information across the neighboring B-scans. Using Layer Normalization instead of Batch Normalization allows training with a batch size of 1. The *pre-activation* strategy [37] ensures that the normalization and *ELU* activations are applied after the skip connections, at the beginning of the next S3DConv block for better gradient backpropagation.

The *Encoder* depicted in Fig. 2(c) has a series of five Basic Encoder Blocks interleaved with downsampling. The Basic Encoder Block comprises two S3DConv Blocks followed by a concatenation based skip connection (see Fig. 2(b)). Here, each S3DConv has *C* input and output channels by setting *P* = *C*/4 in Fig. 2(a). The downsampling is performed with a strided 3×3×3 depthwise-separable convolution [38]. It applies a separate 3×3×3 convolution (with 1 input and output channel) to each of the *C* input channels individually and their outputs are concatenated together. It is implemented in Pytorch by setting *groups* = 1 in the Conv3D layer. Due to large voxel spacing across the B-scans, downsampling along this direction is only performed in the final block with a stride of (2, 2, 2) to ensure a roughly isotropic receptive field. All previous downsampling layers use a (2, 2, 1) stride to only halve the height and width dimensions. The last Encoder block is followed by two parallel pathways, each consisting of two 1×1×1, 3D convolutional layers to obtain the final 64 channel **F**^*D*^ and **F**^*A*^.

##### Decoder

Both *Decoder-D* and *Decoder-A* (in Fig. 1(c)) have the same architecture as shown in Fig. 2(e), except for the number of output channels in the last 1×1×1, convolution layer (1 channel for **A** and 3 to predict **D** respectively). The Decoder architecture employs a series of Basic Decoder Blocks. They map a *C*@*(H, W, D)* input feature map to a $\frac{C}{2}@(2H,2W,D.s)$ output, where *s* is the upsampling factor across B-scans (*s* = 2 in the first block, 1 otherwise). As depicted in Fig. 2(d), it comprises an upsampling layer followed by a S3DConv whose outputs are concatenated with a skip connection. The upsampling layer performs two operations. First, the input of size *C*@*(H, W, D)* is upsampled to *C*@(2*H*, 2*W, D*.*s)* using trilinear interpolation. Next, a Depth-separable 3×3×3 convolution is employed, which divides the *C* input channels into $\frac{C}{4}$ groups of 4 channels each. A separate convolution filter is applied to each group to compress them to a single channel resulting in a $\frac{C}{4}@(2H,2W,D.s)$ output.

### B Downstream TTC Estimation Task

**Fig. 3:**
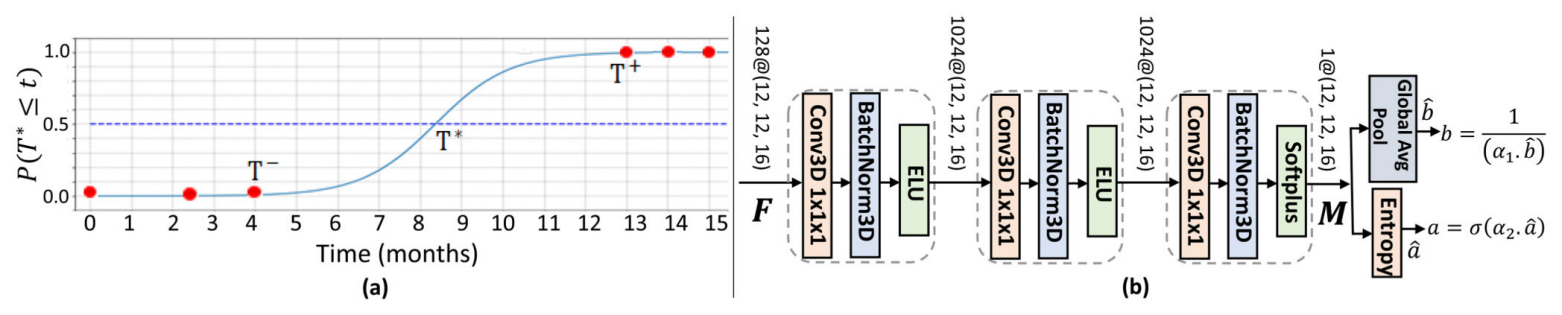
Overview of the TTC Task. **(a)** CDF of the conversion time *T*^∗^ can be best modeled using a sigmoidal function. Exact *T*^∗^ is unknown due to the discrete nature of the visits (red dots) but occurs between the first visit where the eye has converted (*T*^+^) and the visit (*T*^−^) just before it. **(b)** The Classifier Network to predict the parameters of the sigmoidal function.

The problem setting of the Downstream TTC task for an eye is depicted in Fig. 3(a). An OCT is acquired at each visit (red dots) occuring at irregular time intervals. The eye remains in the early/iAMD stage up to the visit at time *T*^−^ and is first diagnosed to have progressed to nAMD at time *T* ^+^. The exact time of conversion *T* ^∗^ is unknown as patients are monitored at discrete time-points but lies in *T*^−^ < *T* ^∗^ ≤ *T* ^+^. We treat *T* ^∗^ as a continuous random variable and aim to model its CDF, *P(T* ^∗^ ≤ *t)* (y-axis in Fig. 3(a)). *P(T* ^∗^ ≤ *t)* is the probability that the eye has converted within the time-point *t*. The binary GT for *P(T* ^∗^ ≤ *t)* is 0 for 0 ≤ *t* ≤ *T*^−^, 1 for *t* ≥ *T* ^+^ and unknown in the range *T*^−^ < *t* < *T* ^+^. We propose to model *P(T* ^∗^ ≤ *t*) with a sigmoidal distribution over time as

(4) $$p_{t}=P(T^{*}\leq t)=1/[1+exp\{-(\frac{t-b}{a+0.05})\}],$$

where *b* is an estimate of *T* ^∗^ and *a* controls the slope of the sigmoidal CDF. A steep slope (small *a*) would indicate a fast progression rate around *T* ^∗^ and viceversa.

#### 1) Classifier Architecture

**Fig. 4:**
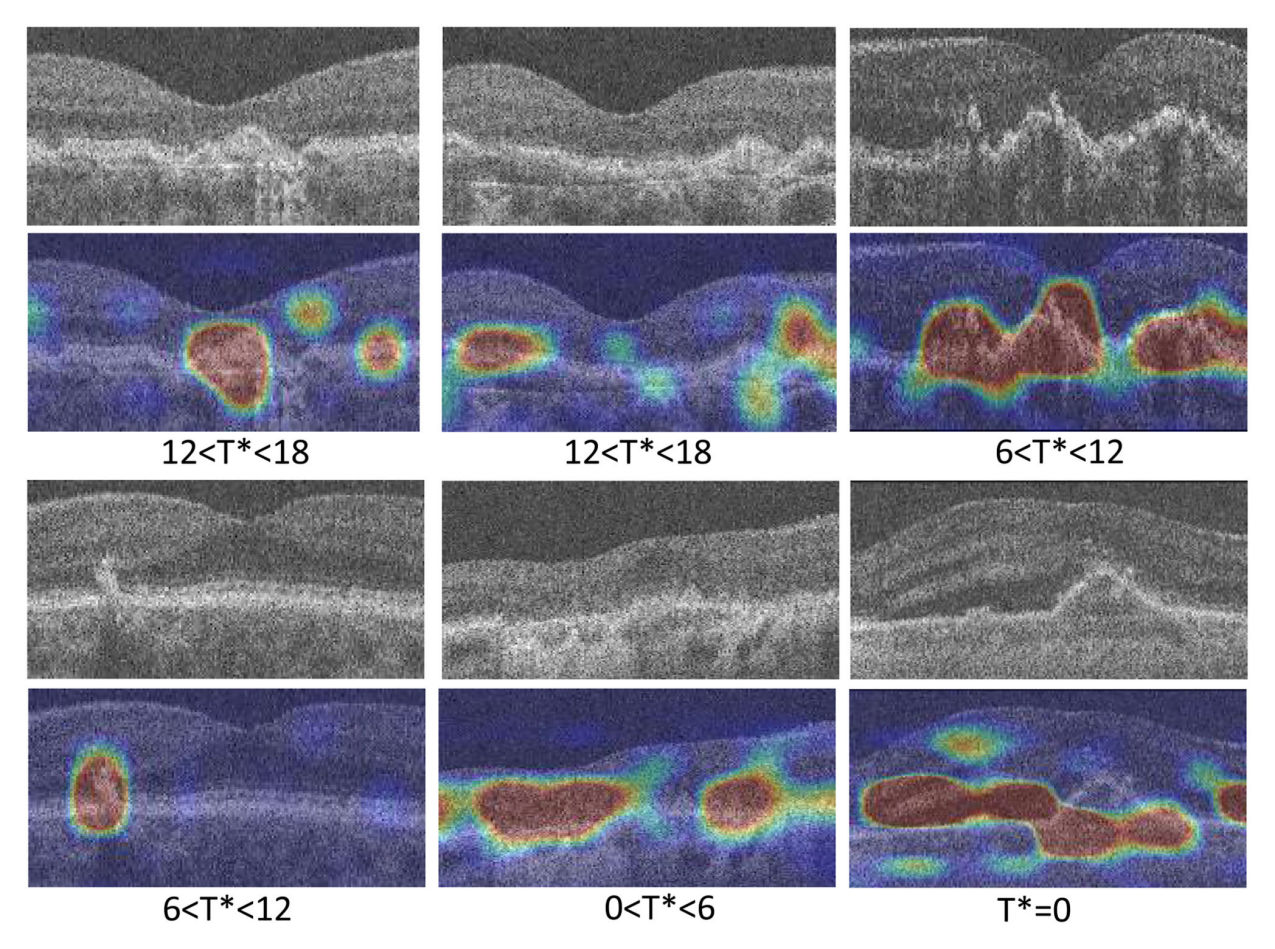
Examples of saliency maps with frozen Morph-SSL weights.

The scalars *a* and *b* are predicted with the classifier in Fig. 3(b). The SSL-trained feature map **F** of the input OCT scan is fed to the classifier. **F** is mapped to a single channel feature map **M** through a series of three 1×1×1 convolutional layers. A Class Activation Map (CAM) can be computed as a weighted sum of all channels in the final convolutional feature map, which in our case is **M** with a single channel. Thus, **M** can be interpreted as a saliency map for our classifier (see Fig. 4) which motivates how *a* and *b* are computed.

The *b* is obtained through the Global Average Pooling (GAP) of **M** denoted by $\hat{b}$, scaling it by a non-negative learnable scalar weight *α*_1_ and taking the reciprocal $b=1/(\alpha_{1}\cdot \hat{b})$. We hypothesize that images predicted to convert soon (with small *b*) should lead to higher activations on the saliency map **M**.

The *a* is obtained by computing the spatial entropy of **M** denoted by $\hat{a}$, scaling it by non-negative learnable scalar *α*_2_ and applying the sigmoid activation. We hypothesize that low entropy (certain locations in **M** have high activations while others take very small values) indicates the detection of some salient regions in the OCT which may correlate to a sudden disease progression around the conversion event leading to a steep slope (small *a*). The spatial entropy is computed by first normalizing **M** to sum to 1, **M**′**(***i*) = **M (***i*) / *∑*_*P*∈Ω_**M**(*p*) and then computing the entropy as *H* = − *∑*_*i*∈Ω_**M**′*(i)*.*log***M**′*(i)*, where Ω represents each spatial position in **M**.

#### 2) Loss Function

A maximum time interval of 18 months (normalized to [0,^1^]) was considered as longer durations are not useful for clinical intervention. The *T* ^∗^ for scans that do not convert within 18 months is unknown. For each scan, the classification loss *ℒ*_*cls*_ consists of the average Binary Cross Entropy loss (BCE) computed for two time-points as

(5) $${ℒ}_{cls}=\{\begin{array}{l} {ℒ}_{ce}(p_{T^{+}},1)+{ℒ}_{ce}(p_{T^{-}},0),\text{if}0\leq T^{+},T^{-}\leq 1\\ {ℒ}_{ce}(p_{0},0)+{ℒ}_{ce}(p_{1},0),\text{if}T^{+},T^{-}>1\\ {ℒ}_{ce}(p_{0},1)+{ℒ}_{ce}(p_{1},1),\text{if}T^{+}=0, \end{array}$$

where *p*_*t*_ at time *t* is computed using Eq. 4. *ℒ*_*ce*_ denotes half of BCE loss to compute the average BCE over the two time-points. The first condition in Eq. 5 occurs when both *T*^−^ and *T* ^+^ occur within 18 months (1 after normalization) and *ℒ*_*ce*_ is computed at these two time-points with the GT labels 0 at *T*^−^ and 1 at *T* ^+^. Since the sigmoidal function is monotonically non-decreasing, minimizing the loss at these two points automatically improves *p*_*t*_ for all *t* because $p_{T^{-}}\approx 0$ also ensures equal or lower predictions before *T* ^–^ and$p_{T^{+}}\approx 0$ enforces equal or higher predictions after *T* ^+^. The second condition in Eq. 5 represents the scenario where the conversion (if the scan ever converts) occurs after 18 months and exact *T* ^+^ and *T*^−^ are unknown. Here, *ℒ*_*ce*_ is computed at *t* = 0 and 1 with a GT label of 0 in both cases. The last condition in Eq. 5 occurs when the input OCT scan is the first visit of conversion and the GT label remains 1 throughout the 18-month interval. In addition to ℒ_*cls*_, two regularization terms are also employed. Thus, the total loss

(6) $${ℒ}_{tot}={ℒ}_{cls}+\gamma_{1}∥a{∥}_{2}^{2}+\gamma_{2}∥\text{M}⊙(1-\hat{\text{R}}){∥}_{1},$$

where *γ*_1_ = *γ*_2_ = 0.1. An L2-regularization of *a* is performed for numerical stability. Moreover, higher activations in **M** outside the retina defined by the binary mask $\hat{\text{R}}$ are penalized. $\hat{\text{R}}$ is the ROI mask of the input scan **R** resized to 12×12×16.

## IV Experiments

### A Dataset

A private longitudinal dataset was created from the Fellow Eyes of a real-world retrospective cohort of OCT scans from the PINNACLE consortium [19] collected from the University Hospital Southampton and Moorfields Eye Hospital. The images were acquired using Topcon scanners with an average 3.6 ± 5.7 months interval between successive visits. A subset of the dataset was manually labelled for the TTC task and the remaining were used to train Morph-SSL.

The *SSL Dataset* had 3570 unlabelled OCT scans from multiple visits of 399 eyes with at least 3 visits per eye. Whenever treatment information was available, the visits after the first anti-VEGF injection were removed to ensure that most scans in the dataset are in the iAMD stage.

The *TTC Dataset* with 343 Eyes (2418 OCT Volumes) was manually examined by clinical experts for the downstream task. In our experiments, each OCT scan was considered independently and the corresponding GT labels for *T* ^+^ (and *T*^−^) were obtained as the time-interval between the current visit and the manually identified first visit of conversion (and the visit just before it). All Scans after the first visit of conversion were removed to focus on the iAMD stage and the earliest indicators of nAMD in the first conversion visit.

### B Preprocessing

The top and bottom boundaries delineating the retinal tissue called the Inner Limiting Membrane (ILM) and the Bruch’s Membrane (BM) were extracted using the automated method in [39]. Thereafter, the curvature of the retinal surface was flattened by shifting each A-scan by an offset such that the BM lies on a straight plane similar to [39]. The binary ROI mask of the retina contained the region from 26 *μm* above the ILM to 169 *μm* (to include the choroid) below the BM. Both the OCT and its ROI mask were then cropped to the central 3×3 *mm*^2^ en-face region. This region has been correlated with the onset of GA and neovascularization [20]. Finally, the volume was resized to 192×192×32 and its intensity linearly scaled to [−1,1].

As an additional preprocessing for the *SSL Dataset*, the enface projections of all visits of an eye were aligned to its first visit using the unsupervised affine registration method in [20]. This step ensures that the Morph-SSL features capture the structural changes caused by AMD progression instead of image misalignment. The step is not performed for the *TTC Dataset* where each visit’s scan is considered independently.

### C Experimental Setup

**Fig. 5:**
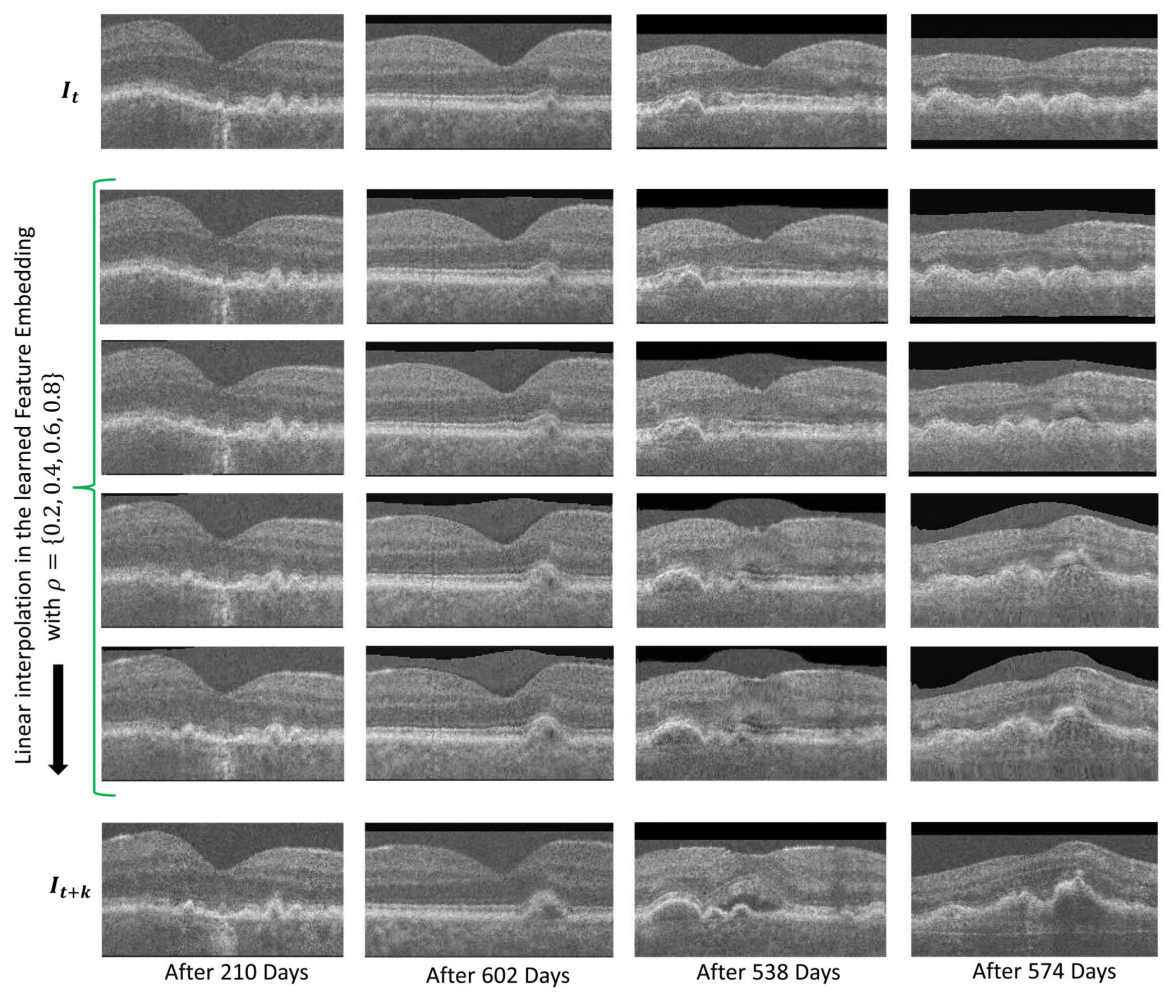
Qualitative visualization of the linear interpolation between the features extracted from two OCT volumes I_***t***_ (first row) and I_*t*+*k*_ of the same eye (last row). A single B-scan from the 3D volume has been depicted for a different eye in each column. The smooth transition in the generated intermediate images demonstrates Morph-SSL’s ability to learn a feature representation with a meaningful notion of distance and direction that correspond to specific morphological changes in the input scan.

Morph-SSL was trained on image pairs formed from two random visits of the same eye, acquired within two years from each other. The *SSL Dataset* was randomly divided into 350 eyes (14078 image pairs) for training, 25 eyes (640 image pairs) for validation and the remaining 24 eyes (600 image pairs) for a qualitative evaluation of the learned features (see Fig. 5).

A stratified five-fold evaluation was conducted for the TTC task to reduce the bias of a specific train-test data split. The *TTC Dataset* was randomly divided into 5 mutually exclusive parts at the eye level. The experiments were repeated 5 times, each time considering one part as the held out test set while the remaining dataset was randomly divided into 85% for training and 15% for validation. The performance was evaluated for predicting the conversion to nAMD within *t* = 0, 6, 12 and 18 months, where t = 0 indicates that the input image is the first visit of conversion. We evaluated prediction scores using the area under the receiver operating characteristic curve (AUC), and we evaluated binary predictions using balanced accuracy calculated as (Sensitivity+Specificity)/2 after thresholding the prediction scores at an operating point that maximized the Youden’s J statistic. We performed both scan-level and eye-level evaluations. The scan-level performance was evaluated on all scans in the test set by treating each patient visit as an independent sample. The average performance across the five-folds is reported in the Appendix, Tables VI-X and the Delong test was employed for statistical significance between AUCs using the pyroc 0.20 library [40]. The eye-level performance assessment employed a *bootstrapping* based approach, incorporating 1000 random eye-level re-samplings of the test set in each fold. Each re-sampling of the test-set contained only one OCT scan (by randomly selecting a patient visit) from each eye. This re-sampling process was repeated 1000 times for each of the five folds, resulting in a total of 5 × 1000 = 5000 sample estimates for each performance metric (AUC and balanced accuracy). The mean and standard deviation across these 5000 sample estimates are reported in Tables I-V and the statistical significance was ascertained with the Wilcoxon Signed Rank Test.

### D Implementation Details

All experiments were implemented in Python 3.8.5 with Pytorch 1.8.1 on a server, using a single NVIDIA A100, 40 GB GPU. An implementation of the proposed method is available at: https://github.com/arunava555/Morph-SSL. Both the Morph-SSL and downstream TTC training employed similar Data Augmentations comprising random 3D translations (up to 15% of the image size along each axis), random horizontal flip (with 0.5 probability), Gaussian blurring (*μ* = 0, random *σ* ∈ [0, 0.9]) and Gaussian noise (*μ* = 0, *σ* = 0.001). For Morph-SSL, both scans in the training image pair were translated and flipped identically, while other augmentations were applied independently. During both training stages, Adam optimizer [41] was used (*β*_1_ = 0.9, *β*_2_ = 0.999, weight decay = 10^−5^ for Morph-SSL, 10^−2^ for TTC) with a cyclic learning rate schedule [42] where the learning rate was linearly varied from *lr*_*min*_ (10^−6^ for Morph-SSL, 10^−5^ for TTC) to *lr*_*max*_ = 10^−4^ and back to *lr*_*min*_ in each epoch. The validation performance was monitored to save the best network weights using minimum loss for Morph-SSL and highest average AUC for TTC.

Morph-SSL trained with a batch size of 1 for 160 epochs, 2000 batch updates per epoch, required 23 GB GPU memory. The downstream training was performed for 400 epochs of 500 batch updates. A batch size of 6 was employed when the Encoder and Classifier were fine-tuned together on the TTC task, requiring 28 GB GPU memory. Training the Classifier alone required 4GB of GPU for a batch size of 16. During inference, the proposed method requires 30.487 GFLOPs and takes an average of 0.04 seconds per image using GPU and 2.2 seconds per image using CPU alone for the downstream conversion prediction task.

During Morph-SSL based pre-training, the size of the encoder’s output and the tunable weights of the loss terms in Eqs. 1 and 3 were empirically fixed by conducting a preliminary hyperparameter search. The large amount of time required in training multiple 3D models prevented a thorough hyperparameter grid search. So, multiple models were trained with different hyperparameter configurations on a small subset of 100 image pairs from the entire *SSL Dataset*. They were manually selected to cover a range of morphological changes, from small to moderate changes in the drusen structure, to large changes in the retinal thickness due to the presence of abnormalities such as PED. Reducing the size of the Encoder’s output feature map below 128@12×12×16 was found to have an adverse impact on the quality of the reconstructed images. Initially, each loss term was scaled in powers of 10 to balance their values to a similar range. Next, the weight of each loss term was varied one at a time in orders of 10 (keeping the other loss weights fixed). The output image reconstructions from the trained models were visually found to provide better image reconstructions when the scale of the loss values were in the following order: ℒ_*prc*_ > deformation term in ℒ_*mse*_ > additive term in ℒ_*mse*_ > ℒ_*f* *ld*_ > ℒ_*add*_ > ℒ_*smth*_. Here, the first three terms guide the network toward better reconstruction, while the weights of the last three regularization terms are kept relatively low to enable it to learn large transformations.

## V Results

### A Results on the TTC Task

#### 1) Impact of Morph-SSL

In Table I, rows 1-3, we evaluate 3 training setups: (a) end-to-end training from random weight initialization (RI); (b) freeze the Morph-SSL trained Encoder weights and only train the classifier on the TTC task (FR); (c) use the Morph-SSL trained Encoder weights and the learned classifier weights from (b) to initialize and perform end-to-end finetuning of the Encoder and Classifier on the TTC task (FN).

The Morph-SSL features showed significant performance improvement, even without fine-tuning, over end-to-end training form scratch (row 1 vs 3). Further fine-tuning on the TTC task (row 1 vs. 2) did not lead to a statistically significant performance improvement, except for *t* = 18. This indicates that the initial Morph-SSL trained weights are very close to the optimal network weights for the TTC task. Overall, a good performance is observed in identifying the scans that have just converted to nAMD (*t* = 0) or are about to convert within 6 months. However, the AUC drops progressively as we consider larger time-intervals for forecasting into the future. This may indicate that often, distinct morphological changes signaling imminent nAMD conversion appear unexpectedly only a few months before conversion rather than gradually over a long period. Similar trends are also observed for the scan-level performance reported in the Appendix, Table VI.

A few examples of the Saliency Maps **M** of the proposed method are shown in Fig. 4 for OCT scans that convert at different time intervals in the future. It shows that the trained model is sensitive to abnormalities in the outer retina such as drusen, PED and HRF, which are known to be associated with AMD progression [2], [16], [17].

#### 2) Comparison With TTC regression

Conversion prediction can also be posed as a regression task for predicting the TTC. In order to compare our approach against regression, we use the same Encoder (pre-trained with Morph-SSL) and the downstream classification network architecture (in Fig. 3(b)) but the prediction obtained through GAP of the final single channel output **M** is treated as the time-to-conversion from the current visit. The GT was computed as the mean of *T*^−^ and *T* ^+^ and used for training with a MSE loss. The output prediction is binned into 6-month intervals to obtain the binary prediction of nAMD conversion within *t* = 0, 6, 12, 18 months for comparison with our method (see row 4,5 in Table I for eye-level and Appendix, Table VI for scan-level performance). Since, the resulting predictions are binary and not continuous scores, AUC could not be computed and only balanced accuracy has been reported. A large drop in performance is observed between this regression-based approach as compared to our proposed method of modeling the CDF (with a sigmoid function over time) trained with the BCE loss at different time-points in Eq. 5. This performance gap can be primarily attributed to the inability of the regression-based approach to utilize training samples that do not convert within the time-period of 18 months as their GT label for the conversion time is unknown while these samples are also used for training in our formulation (second condition in Eq. 5). Another problem with the regression-based formulation is the unavailability of the exact conversion date *T* * between *T* ^+^ and *T*^−^ leading to noisy labels. Furthermore, in some cases, formulating regression problems as a classification task has been shown to yield better performance. This has been linked to the ability of the cross-entropy-based classification loss to learn high-entropy (more diverse) feature representations as compared to regression with a MSE loss [43].

#### 3) Impact of the Encoder Architecture

In this work, we propose an efficient CNN for 3D input images that optimizes the amount of computation and trainable parameters. Our solution involves two modifications: (i) substituting 3 × 3 × 3, 3D convolutions with S3DConv which applies 3 × 3 convolutions along the three orthogonal spatial orientations (see Fig. 2(a)); (ii) using concatenation-based skip connections [44] instead of the additive residual skip connections in the Basic Blocks in Fig. 2(b), (d) which are used at each scale of our Encoder and Decoder architectures. We also employ Layer Normalization instead of Batch Normalization which enables stable training with small batch sizes on limited GPU memory. Substituting 3 × 3 × 3 convolutions with S3DConv in our Encoder-Decoder architecture reduces computation from 189 GFLOPs (with full 3D convolutions) to 71 GFLOPs and the 6,622,457 trainable network parameters to 2,702,329 during the Morph-SSL based pre-training. To evaluate the impact of the concatenation-based skip connections, the proposed Basic Encoder and the Basic Decoder Block in Fig. 2(b), (d) were substituted with the additive residual connection-based alternatives depicted in the Appendix, Fig. 7(a), (b). These modified Basic Encoder and Decoder Blocks require double the number of convolutional filters in the two S3DConv layers inside them compared to our concatenation-based skip connections for the same number of input and output channels, thereby requiring significantly more computation (203G vs 71G FLOPs) and network parameters (7,025,881 vs 2,702,329).

Although full 3D convolutions require a significant amount of computation and network parameters, S3DConv performs better in both settings with frozen Morph-SSL pre-trained weights (rows 1,2 in Table II) and fine-tuning (rows 5,6 in Table II) at all time-points. Full 3D convolutions are more prone to over-fitting when trained on limited labelled data for the downstream conversion prediction task. The performance of Layer Normalization (pre-trained with a batch size of 1) and Batch Normalization (pre-trained with a batch size of 2, limited by GPU memory) is presented in Table II (rows 1,3 for frozen Morph-SSL pre-trained weights and rows 3,5 with fine-tuning). Overall, Layer Normalization outperforms Batch Normalization under both settings at *t* = 0, 6, 12 whereas Batch Normalization performs better at *t* = 18. The proposed concatenation-based skip connections also outperform the residual additive skip connections for both the frozen and fine-tuned models with a statistically significant difference at all time-points (rows 1,4 and rows 5,8 in Table II). Our architectural choices also exhibited better performance in the scan-level evaluations reported in Appendix, Table VII.

#### 4) Impact of the Loss Terms for the TTC Task

An ablation of the auxiliary loss terms in Eq. 6 is evaluated in rows 2, 3 of Table III at an eye-level (and Table VIII at a scan-level). The removal of L-2 regularization on the slope parameter *a* (row 1 vs 2) causes a minor drop in the AUC and Balanced Accuracy across all time points except *t* = 0, where no statistically significant difference is observed.

Removing the loss term which penalizes high activations outside the retinal tissue leads to a small drop in both AUC and Balanced Accuracy for all time-points (row 3 vs 1). However the difference at *t* = 18 was not statistically significant. Similar overall trends are also observed in the scan-level performance reported in the Appendix, Table VIII.

#### 5) Impact of Our TTC Formulation

We propose to model the CDF of the TTC with a sigmoidal function. An alternative way is to pose it as multi-label classification with each class indicating if the image converts within a discrete time-point [13], [14], [30]. We compare our eye-level performance against multi-label classification in Table III, row 4 by modifying the last layer of our classifier architecture to produce a 4 channel ouptut (instead of 1), to which GAP is applied followed by a sigmoid activation to obtain the predictions for the 4 time-points. Both in terms of AUC and the Balanced accuracy, the proposed method clearly outperforms multi-label classification at *t* = 12, 18. At *t* = 6, the slightly better performance of our method was not statistically significant while the multi-label classification performed better at *t* = 0. Similar performance trends are also observed at the scan-level in the Appendix, Table VIII row 4. Although the performance of both methods are similar, our approach guarantees the monotonic increasing property of the CDF (e.g., the probability of an eye to convert within 12 months cannot be lower than the conversion probability within 6 months) which is not the case with multi-label classification. Across the 5 folds, the multi-label classifier is inconsistent in some cases, with higher prediction scores for a previous time-point compared to the next for a given input scan, 16 cases between *t* = 0, 6 months, 60 cases between *t* = 6, 12 and 84 cases with inconsistencies between *t* = 12, 18 months. Additionally, once trained, our model can predict conversion risk at any time-point within 18 months by varying *t* in eq 4, unlike multi-label classification that can predict conversion risk only at predefined discrete time intervals used during training.

#### 6) Architecture Design to Predict Slope

Spatial entropy of **M** was used to predict the slope *a* of the sigmoid function. We compared this design choice against one which predicts two channels. One channel is used similar to **M** to compute *b* while a GAP is applied to the second channel for obtaining *a*. Although this new architecture requires extra network parameters, it did not result in a statistically significant difference in performance (see rows 1, 5 in Table III).

#### 7) Comparison With State-of-the-Art 3D Networks

An alternative to SSL is to fine-tune standard CNN networks after initializing them with the already available pre-trained weights. We compared our performance against two popular 3D-CNN networks, I3D [45] and X3D [46]. Their last fully connected layer was modified to predict *a* and *b* in Eq. 4. Both networks were initialized with pre-trained weights trained on the Kinetics video dataset and fine-tuned end-to-end on our task. Our Morph-SSL trained Encoder significantly outperformed both of these networks (see rows 1-3 in Table IV for eye-level and Appendix, Table IX for scan-level performance) in terms of both AUC and Balanced accuracy across all time-points.

#### 8) Comparison With Handcrafted Biomarker Based Method

Recent clinical research has linked the spatial distribution of drusen and HRF to the future progression of AMD [16], [17]. Similar to [17], we segmented the drusen and HRF automatically using the Iowa Reference Algorithm [39] with modified smoothness constraints [47] for drusen and a deep learning-based method [48] for HRF. Thereafter, the volume of drusen, HRF, and their areas in the enface (surface) projection were computed in 14 spatial sectors (entire scan, central 1 mm disc, central 3 mm disc, central 6 mm disc, peri-fovea, para-fovea, peri-nasal, para-nasal, peri-superior, para-superior, peri-inferior, para-inferior, peri-temporal and para-temporal sectors) based on the ETDRS grid commonly used in clinical research. This resulted in a 14 (sectors) × 4 (area and volume of HRF and drusen) = 56 dimensional feature vector. A multi-output random forest comprising 350 decision trees (selected based on best validation performance) was trained to predict the conversion to nAMD for the different time-points. The results presented in Table IV (and Appendix, Table IX), rows 1 and 4, indicate the superiority of the proposed method over handcrafted biomarkers. This highlights the inadequacy of the current clinically known prognostic features in predicting nAMD conversion and motivates the use of DL networks that directly learn the prognostic imaging features from raw OCT scans instead of hand-crafting them.

#### 9) Comparison With Other SSL Methods

We compare the performance of Morph-SSL against other state-of-the-art SSL methods at an eye-level in Table V and scan-level in Appendix, Table X. The same 3D U-net and the transformations for the reconstruction task were employed for Model Genesis as reported in [3]. The time interval prediction task [14] was originally developed for the central B-scans alone, however we implemented a 3D version using our Encoder architecture for a fair comparison. The latest CL methods, VICReg [9] and Barlow Twins [10] could not be trained in 3D due to their large batch size requirements. They were used to train a ResNet-50 with a batch size of 128 following [13]. The positive image pairs were constructed by selecting B-scans (from the same position) from two random visits of the same eye within 18 months and applying the data augmentations used in [13]. The Classifier was modified to handle a 2048×32 (feature dimensions × B-scans) input. First, a 1D convolution layer with 32 input and 1 output feature channel was used to obtain a 2048 dimensional feature for the entire OCT volume. This was followed by two fully connected layers with 1024 and 2 neurons respectively, to get the predictions for *a* and *b* in Eq. 4. The SSL methods were compared under different training setups by: (a) using the SSL-trained features off-the-shelf and only training the Classifier (Freeze) vs. initialization with the SSL-trained network weights for end-to-end fine-tuning (Finetune), and (b) training on the entire vs. one-third of the supervised training data. To evaluate performance in a small data regime, one-third of the training data in each fold of the *TTC Dataset* was randomly selected and kept consistent across all SSL methods.

##### Small Data Regime

Morph-SSL outperforms all benchmark methods (under identical Freeze/Finetune setup) across all 4 time-points in Table V. All differences were statistically significant. Overall, finetuning improves performance over frozen weights for all SSL methods except for Model-Genesis at *t* = 0, 18 and Barlow Twins at *t* = 0, 6.

##### Entire Training Data

In the Freeze setup, again Morph-SSL outperforms all benchmark methods in terms of AUC with a statistically significant difference across all 4 time-points. In the fine-tuning setup, Morph-SSL still clearly outperforms time-interval prediction [14], Barlow twins [10] and VICReg [9] across all time-points. Although Morph-SSL outperformed Model-Genesis at *t* = 0, 6, the AUC difference was not statistically significant for *t* = 12 (p-value 0.43), while Model-Genesis performed better at *t* = 18.

Overall, Morph-SSL shows better performance than other methods, particularly in scenarios where features are used off-the-shelf or in a small data regime with limited labeled data for fine-tuning. When trained on the entire dataset, Morph-SSL was found to learn strong features with good performance on the TTC task with minimal effect of further fine-tuning.

### B Risk Score for Progression to nAMD

**Fig. 6:**
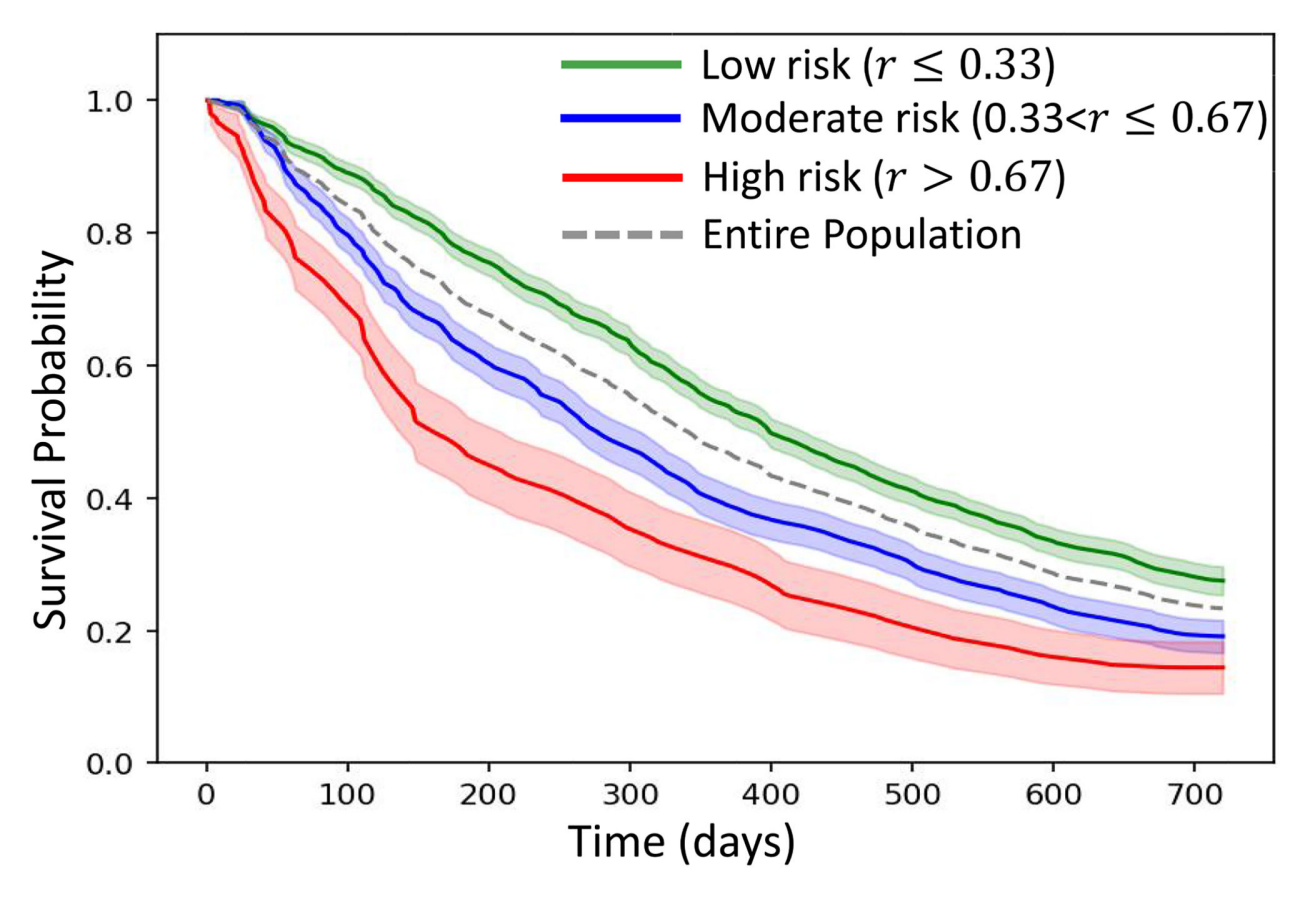
Kaplan-Meier curves for different risk groups.

As part of current clinical practice, patients with iAMD undergo regular eye examinations and nAMD must be treated at the earliest sign of onset to prevent vision loss. However, biomarkers such as drusen volume and hyper-reflective foci (HRF) are insufficient to reliably identify the patients at a higher risk of conversion. As a result, an automated method to stratify iAMD eyes into low and high risk groups for future conversion to nAMD can help clinicians prioritize patients in the high risk group for early treatment and frequent monitoring. An ideal risk score should a) be a single time-independent scalar value; b) be bounded in the range [0, 1]; c) be inversely proportional to the predicted time to conversion *b*. We formulate such a risk score by modifying Eq. 4 as $r=2/[1+exp\{\frac{b}{a+0.05}\}]$. The test predictions for *a* and *b* were obtained from the five folds to compute *r* for each OCT scan. The scans were then stratified into 3 groups with low risk (0 ≤ *r* ≤ 0.33), moderate risk (0.33 < *r* ≤ 0.67) and high risk (0.67 < *r* ≤ 1). A population-level survival function for these groups is plotted in Fig. 6 using the Kaplan–Meier estimator on the GT conversion time. It depicts the mean and standard deviation of the survival probability for each group, computed across 1000 re-samplings using eye-level bootstrapping. Each scan within a re-sampling was independent and came from a different eye as only one OCT scan (from a random visit) was selected per eye during bootstrapping. A log-rank t-test between the curves was performed in a pairwise manner among the three risk groups and the median p-value across all bootstrap re-samplings was used to determine the statistical significance. The difference between the survival curves of the low-risk and the high-risk groups was found to be highly statistically significant with a p-value of 0.007. The difference between the medium-risk vs. the high-risk group was not significant (p-value= 0.263) while the difference between the low-risk and the medium-risk groups was also significant with a p-value< 0.05 (p-value= 0.034). Overall, *r* is effective in stratifying eyes coming from low and high risk groups.

#### 1) Intra-Eye Consistency

AMD is a degenerative disease where the retinal tissue progressively deteriorates, so the predicted risk scores from scans across multiple visits of the same patient should be monotonically increasing over time. However, this consistency is not explicitly enforced by the downstream classifier for conversion prediction which uses single OCT scans as input and treats each image as an independent sample (although the Morph-SSL pretraining employs pairs of visits to learn the feature embedding). Therefore to quantitatively assess the intra-eye consistency in predicted risk scores, we computed the eye-level concordance index (eCI). It involved constructing a set of all possible pairs of visits (**I**_*t*_, **I**_*t*+*k*_) for each eye. Each scan was independently fed into our trained model to derive the corresponding pair of risk scores *(r*_*t*_, *r*_*t*+*k*_). The eCI was then calculated as the fraction of the visit pairs (out of the total number of all possible pairs for the eye), in which the predicted risk scores adhered to the desired ordering *r*_*t*+*k*_ ≥ *r*_*t*_. Despite that the classifier treated each eye as an independent sample, the average eCI across all eyes in all folds was 0.73, indicating a moderately good consistency in the risk score predictions.

### C Interpolation in the Morph-SSL Feature Space

Given a pair of scans **I**_*t*_, **I**_*t*+*k*_ from two visits of the same eye, we extract their features **F**_*t*_ and **F**_*t*+*k*_, and generate an intermediate feature through linear interpolation as ${\text{F}}_{\rho }^{\prime }={\text{F}}_{t}+\rho \cdot ({\text{F}}_{t+1}-{\text{F}}_{t})$, where *ρ* ∈ [0, 1]. By using **F**_*t*_ and ${\text{F}}_{\rho }^{\prime }$ (instead of **F**_*t*+*k*_) as inputs to the Morph-SSL trained Decoder, we can predict the transformation that morphs **I**_*t*_ to artificially generate the intermediate OCT scan for ${\text{F}}_{\rho }^{\prime }$ (see Fig. 1(c)). The qualitative results in Fig. 5 depict four intermediate scans (along each column) by varying *ρ*. A gradual smooth transition between **I**_*t*_ and **I**_*t*+*k*_ is observed with the generated scans. Such a smooth feature embedding is enforced by our Decoder architecture which explicitly correlates the direction of the feature displacement ${\text{F}}_{\rho }^{\prime }-{\text{F}}_{t}$ to the *type*, and its magnitude to the *amount* of the morphing transformation. The magnitude increases with *ρ* while the direction remains the same.

This property may be explored in the future for different applications. Balanced-Mixup [49] generates artificial training samples by directly interpolating the voxels between two training images, which may produce blurry images. Through small interpolations in our feature embedding instead, better training samples may be generated. Another potential application could be to generate approximations of future OCT scans to visualize disease progression. A Recurrent Neural Network to predict the features for future visits may be explored for this task.

## VI Conclusion

A vast amount of unlabelled longitudinal OCT scans are generated in clinics to monitor AMD. To leverage this data, we have proposed Morph-SSL, a novel SSL method designed to capture the temporal changes caused by disease progression. It ensures that the displacement in features between two OCT scans captures the morphological changes in the retina between them. With the Morph-SSL trained Encoder, we have developed a prognostic model for TTC estimation that predicts the future risk of conversion from iAMD to nAMD from the current OCT scan. The lack of reliable biomarkers and wide variability in the rate of AMD progression makes it a challenging task. We modelled the CDF of TTC with a sigmoidal function over time. The Morph-SSL features were found to perform well on the TTC task even without fine-tuning and showed significant improvements over training from scratch or fine-tuning standard 3D-CNNs with pre-trained weights. It also outperformed popular SSL methods with significant gains in scenarios where SSL features are used off-the-shelf or fine-tuned on limited labeled data. We also derived a risk score that could be used to stratify eyes into low or high risk categories. Identifying iAMD patients with a high risk of progressing to nAMD can enable ophthalmologists to prioritize these cases for closer monitoring. Initiating treatment at the earliest sign of nAMD onset is crucial to prevent irreversible vision loss. Thus, our method to forecast the risk of future AMD progression can play a critical role in enabling patient-specific disease management and also aid in enriching clinical trial populations through the recruitment of patients at risk.

### A Limitations and Future Directions

The large amount of time required for training multiple 3D CNN networks prevented an exhaustive search for the optimal network architecture, the size of Encoder’s output feature map, and the tunable weights of the loss terms used during Morph-SSL training, which remains a limitation of this work. Currently, the classification network for forecasting the conversion risk treated each scan acquired at different time-points of the same eye as independent training samples. Although the current model showed a moderate amount of consistency between predictions from different visits for the same future time-point (eCI = 0.73), an alternate approach for the supervised downstream task training may be explored in the future to explicitly enforce this consistency constraint. Finally, although Morph-SSL was primarily developed to pre-train the Encoder in an unsupervised manner, the learned Encoder-Decoder network can additionally smoothly interpolate between the scans from two visits. This offers promising future research directions for using the interpolated scans as a data augmentation or to visualize the expected future morphological changes if a Recurrent Neural Network could be trained to predict the feature representations of future visits. Adapting Morph-SSL to other prognostic tasks in the medical domain, such as forecasting cancer progression from ultrasound images or predicting the future onset of dementia from MRI scans, offers important directions for future work.

## Supplementary Material

Appendix

## Figures and Tables

**Table I:** EYE-LEVEL EVALUATION (MEAN±STD. DEVIATION) OF MORPH-SSL PRE-TRAINING AFTER FREEZING WEIGHTS (FR) AND END-TO-END FINE-TUNING (FN), COMPARED WITH RANDOM NETWORK INITIALIZATION (RI). THE PROPOSED METHOD OF MODELING THE TIME-TO-CONVERSION IS COMPARED AGAINST REGRESSION IN ROWS 4-5. THE VALUES HIGHLIGHTED WITH * ARE **N****OT** STATISTICALLY DIFFERENT FROM PROPOSED-FR (ROW 1) WITH *p* > 0.05

SL. No.		0 month		6 month		12 month		18 month	
		AUC	Bal Acc.	AUC	Bal Acc.	AUC	Bal Acc.	AUC	Bal Acc.
1	Proposed-FR	**0.856 ± 0.05**	0.817 ± 0.05	0.777 ± 0.05	0.753 ± 0.04	0.733 ± 0.07	0.729 ± 0.05	0.712 ± 0.09	0.728 ± 0.07
2	Proposed-FN	0.851 ± 0.05	0.818 ± 0.05*	0.779 ± 0.05*	**0.758 ± 0.04**	0.734 ± 0.07*	**0.735 ± 0.05**	**0.719 ± 0.09**	**0.737 ± 0.07**
3	Proposed-RI	0.785 ± 0.08	0.763 ± 0.07	0.711 ± 0.06	0.711 ± 0.05	0.688 ± 0.08	0.701 ± 0.06	0.676 ± 0.1	0.702 ± 0.07
4	Regression-FR	—	0.681 ± 0.06	—	0.660 ± 0.04	—	0.633 ± 0.07	—	0.583 ± 0.08
5	Regression-FN	—	0.706 ± 0.08	—	0.680 ± 0.07	—	0.654 ± 0.07	—	0.589 ± 0.08

**Table II:** EYE-LEVEL PERFORMANCE (MEAN ± STD.DEV) FOR ABLATION ON THE ENCODER-DECODER ARCHITECTURE. EACH NETWORK IS PRE-TRAINED WITH MORPH-SSL AND EVALUATED BY EITHER FREEZING (FR) WEIGHTS OR END-TO-END FINETUNING (FN) ON THE DOWNSTREAM TASK. THE BEST VALUE IN EACH COLUMN IS HIGHLIGHTED IN BOLD. THE STATISTICAL SIGNIFICANCE OF ROWS 2-4 IS COMPARED WITH ROW 1 AND ROWS 6-8 WITH ROW 5 AND THE VALUES HIGHLIGHTED WITH * ARE **N****OT** STATISTICALLY DIFFERENT WITH *p* > 0.05

SL. No.		0 month		6 month		12 month		18 month	
		AUC	Bal Acc.	AUC	Bal Acc.	AUC	Bal Acc.	AUC	Bal Acc.
1	Proposed-FR	**0.856 ± 0.05**	0.817 ± 0.05	0.777 ± 0.05	0.753 ± 0.04	0.733 ± 0.07	0.729 ± 0.05	0.712 ± 0.09	0.728 ± 0.07
2	3D Convolutions-FR	0.840 ± 0.06	0.804 ± 0.06	0.760 ± 0.06	0.740 ± 0.05	0.718 ± 0.08	0.720 ± 0.06	0.705 ± 0.09	0.721 ± 0.07
3	BatchNorm-FR	0.842 ± 0.05	0.811 ± 0.05	0.756 ± 0.05	0.740 ± 0.04	0.724 ± 0.06	0.726 ± 0.05*	0.719 ± 0.08	0.734 ± 0.06
4	Additive skip conn-FR	0.829 ± 0.06	0.794 ± 0.05	0.753 ± 0.05	0.738 ± 0.04	0.713 ± 0.07	0.716 ± 0.05	0.688 ± 0.08	0.711 ± 0.06
5	Proposed-FN	0.851 ± 0.05	**0.818 ± 0.05**	**0.779 ± 0.05**	**0.758 ± 0.04**	**0.734 ± 0.07**	**0.735 ± 0.05**	0.719 ± 0.09	**0.737 ± 0.07**
6	3D Convolutions-FN	0.844 ± 0.05	0.807 ± 0.05	0.763 ± 0.05	0.740 ± 0.04	0.722 ± 0.08	0.722 ± 0.05	0.708 ± 0.09	0.723 ± 0.07
7	BatchNorm-FN	0.834 ± 0.06	0.802 ± 0.05	0.757 ± 0.05	0.741 ± 0.04	0.727 ± 0.07	0.728 ± 0.05	0.722 ± 0.07*	0.735 ± 0.05*
8	Additive skip conn-FN	0.823 ± 0.06	0.792 ± 0.06	0.754 ± 0.06	0.738 ± 0.05	0.714 ± 0.08	0.721 ± 0.05	0.688 ± 0.09	0.713 ± 0.06

**Table III:** EYE-LEVEL PERFORMANCE (MEAN ± STD. DEVIATION) FOR ABLATION ON THE DOWNSTREAM TTC CLASSIFICATION LOSS (ROWS 2-3) AND THE CLASSIFIER ARCHITECTURE (ROWS 4-5). THE MORPH-SSL PRE-TRAINED ENCODER WEIGHTS ARE FROZEN AND ONLY THE CLASSIFIER IS TRAINED IN EACH EXPERIMENT. THE BEST VALUE IN EACH COLUMN IS HIGHLIGHTED IN BOLD. ROWS 2-5 ARE COMPARED WITH ROW 1 AND THE VALUES WHICH ARE **N****OT** STATISTICALLY SIGNIFICANT (*p* > 0.05) ARE HIGHLIGHTED WITH A *

SL. No.		0 month		6 month		12 month		18 month	
		AUC	Bal Acc.	AUC	Bal Acc.	AUC	Bal Acc.	AUC	Bal Acc.
1	Proposed	0.856 ± 0.05	0.817 ± 0.05	**0.777 ± 0.05**	**0.753 ± 0.04**	**0.733 ± 0.07**	0.729 ± 0.05	**0.712 ± 0.09**	**0.728 ± 0.07**
2	no $∥a{∥}_{2}^{2}$	0.852 ± 0.05*	0.818 ± 0.05*	0.765 ± 0.05	0.746 ± 0.04	0.723 ± 0.08	0.722 ± 0.05	0.699 ± 0.09	0.717 ± 0.07
3	no $∥M⊙(1-\hat{R}){∥}_{1}$	0.845 ± 0.06	0.811 ± 0.05	0.762 ± 0.06	0.741 ± 0.05	0.723 ± 0.08	0.721 ± 0.06	0.707 ± 0.09*	0.727 ± 0.07*
4	Multilabel Classifier	**0.879 ± 0.05**	**0.842 ± 0.05**	0.774 ± 0.06*	0.752 ± 0.05*	0.724 ± 0.07	0.722 ± 0.05	0.700 ± 0.08	0.718 ± 0.07
5	Separate *a* prediction	0.853 ± 0.05*	0.816 ± 0.06*	0.776 ± 0.05*	0.751 ± 0.04*	0.732 ± 0.07*	0.730 ± 0.05*	0.710 ± 0.08*	0.727 ± 0.06*

**Table IV:** EYE-LEVEL PERFORMANCE (MEAN±STD. DEVIATION) TO BENCHMARK THE PROPOSED METHOD (FINE-TUNED FROM MORPH-SSL PRE-TRAINED WEIGHTS) AGAINST STANDARD 3D NETWORKS (FINE-TUNED FROM THEIR AVAILABLE WEIGHTS PRE-TRAINED ON THE KINETICS DATASET) AND HANDCRAFTED BIOMARKERS WITH A RANDOM FOREST CLASSIFIER. THE BEST PERFORMANCE IN EACH COLUMN IS HIGHLIGHTED IN BOLD. THE PERFORMANCE DIFFERENCE OF ROWS 2-4 COMPARED TO THE PROPOSED METHOD (ROW 1) WAS FOUND TO BE STATISTICALLY SIGNIFICANT WITH (*p* < 0.05) FOR ALL TIME-POINTS

SL. No.		0 month		6 month		12 month		18 month	
		AUC	Bal Acc.	AUC	Bal Acc.	AUC	Bal Acc.	AUC	Bal Acc.
1	Proposed-Finetune	**0.851 ± 0.05**	**0.818 ± 0.05**	**0.779 ± 0.05**	**0.758 ± 0.04**	**0.734 ± 0.07**	**0.735 ± 0.05**	**0.719 ± 0.09**	**0.737 ± 0.07**
2	I3D [45]	0.784 ± 0.08	0.776 ± 0.06	0.715 ± 0.07	0.716 ± 0.05	0.672 ± 0.07	0.693 ± 0.05	0.667 ± 0.08	0.697 ± 0.06
3	X3D [46]	0.774 ± 0.07	0.769 ± 0.06	0.710 ± 0.06	0.716 ± 0.05	0.682 ± 0.07	0.700 ± 0.05	0.679 ± 0.08	0.710 ± 0.06
4	Biomarkers+Random Forest	0.752 ± 0.07	0.738 ± 0.05	0.695 ± 0.07	0.699 ± 0.06	0.664 ± 0.08	0.678 ± 0.06	0.629 ± 0.09	0.666 ± 0.06

**Table V:** EYE-LEVEL AREA UNDER THE ROC CURVE (MEAN±STD. DEVIATION) TO COMPARE SSL METHODS UNDER DIFFERENT TRAINING CONFIGURATIONS BY: EITHER TRAINING ON ONE-THIRD OR THE ENTIRE TRAINING DATASET; EITHER FREEZING SSL-TRAINED WEIGHTS OR FINETUNING END-TO-END. THE VALUES HIGHLIGHTED WITH * IN EACH COLUMN ARE **N****OT** STATISTICALLY DIFFERENT (*p* > 0.05) COMPARED TO THE PROPOSED METHOD TRAINED WITH IDENTICAL DATA (EITHER ONE-THIRD OR THE ENTIRE DATASET) AND PROTOCOL (EITHER FREEZE SSL WEIGHTS OR FINETUNE). THE BEST PERFORMANCE IN EACH COLUMN IS HIGHLIGHTED IN **B****OLD**

	One-third Training data				Entire Training data			
	0 month	6 month	12 month	18 month	0 month	6 month	12 month	18 month
Proposed-Freeze	0.835 ± 0.06	0.764 ± 0.06	0.727 ± 0.08	0.705 ± 0.10	**0.856 ± 0.05**	0.777 ± 0.05	0.733 ± 0.07	0.712 ± 0.09
Proposed-Finetune	**0.846 ± 0.05**	**0.780 ± 0.06**	**0.729 ± 0.07**	**0.714 ± 0.09**	0.851 ± 0.05	**0.779 ± 0.05**	**0.734 ± 0.07**	0.719 ± 0.09
Model Genesis-Freeze [3]	0.785 ± 0.07	0.712 ± 0.06	0.671 ± 0.07	0.663 ± 0.08	0.801 ± 0.06	0.718 ± 0.06	0.680 ± 0.07	0.668 ± 0.09
Model Genesis-Finetune [3]	0.772 ± 0.06	0.716 ± 0.07	0.688 ± 0.09	0.661 ± 0.10	0.823 ± 0.06	0.763 ± 0.05	0.733 ± 0.07*	**0.728 ± 0.09**
Time prediction-Freeze [14]	0.586 ± 0.07	0.557 ± 0.08	0.527 ± 0.09	0.522 ± 0.11	0.690 ± 0.09	0.605 ± 0.08	0.575 ± 0.10	0.567 ± 0.12
Time prediction-Finetune [14]	0.626 ± 0.13	0.610 ± 0.12	0.582 ± 0.11	0.561 ± 0.12	0.735 ± 0.08	0.669 ± 0.07	0.644 ± 0.07	0.608 ± 0.09
Barlow Twins-Freeze [10]	0.765 ± 0.06	0.687 ± 0.06	0.630 ± 0.08	0.598 ± 0.10	0.742 ± 0.07	0.692 ± 0.07	0.667 ± 0.07	0.654 ± 0.09
Barlow Twins-Finetune [10]	0.747 ± 0.07	0.671 ± 0.07	0.656 ± 0.08	0.643 ± 0.10	0.749 ± 0.08	0.686 ± 0.07	0.669 ± 0.08	0.648 ± 0.09
VICReg-Freeze [9]	0.767 ± 0.07	0.710 ± 0.06	0.688 ± 0.07	0.674 ± 0.07	0.817 ± 0.05	0.744 ± 0.05	0.713 ± 0.06	0.688 ± 0.07
VICReg-Finetune [9]	0.808 ± 0.06	0.741 ± 0.06	0.711 ± 0.06	0.703 ± 0.07	0.824 ± 0.06	0.750 ± 0.05	0.714 ± 0.07	0.698 ± 0.08
