# Supplementary material for: Morph-SSL: Self-Supervision With Longitudinal Morphing for Forecasting AMD Progression From OCT Volumes
Source: IEEE Trans Med Imaging. Author manuscript; Available in PMC 2024 Oct 14. (PMC7616690; doi:10.1109/TMI.2024.3390940)
Supplement: Appendix [file EMS198503-supplement-Appendix.pdf]

TABLE VI

SCAN-LEVEL EVALUATION (MEAN±STD. DEVIATION) OF MORPH-SSL PRE-TRAINING AFTER FREEZING WEIGHTS (FR) AND END-TO-END FINE-TUNING (FN), COMPARED WITH RANDOM NETWORK INITIALIZATION (RI). THE PROPOSED METHOD OF MODELING THE TIME-TO-CONVERSION IS COMPARED AGAINST REGRESSION IN ROWS 4-5. THE AUC VALUES HIGHLIGHTED WITH * ARE **NOT** STATISTICALLY DIFFERENT FROM PROPOSED-FR (ROW 1) WITH $p > 0.05$

SL.		0 month		6 month		12 month		18 month	
No.		AUC	Bal Acc.	AUC	Bal Acc.	AUC	Bal Acc.	AUC	Bal Acc.
1	Proposed-FR	$\mathbf{0.876 \pm 0.02}$	0.810 ± 0.02	0.767 ± 0.02	0.721 ± 0.01	0.718 ± 0.04	0.686 ± 0.02	0.693 ± 0.06	0.673 ± 0.04
2	Proposed-FN	$0.871 \pm 0.02^*$	$\mathbf{0.812 \pm 0.02}$	$\mathbf{0.768 \pm 0.02}^*$	$\mathbf{0.727 \pm 0.01}$	$\mathbf{0.721 \pm 0.05}^*$	$\mathbf{0.691 \pm 0.03}$	$\mathbf{0.700 \pm 0.06}$	$\mathbf{0.674 \pm 0.05}$
3	Proposed-RI	0.799 ± 0.04	0.742 ± 0.04	0.704 ± 0.03	0.673 ± 0.02	0.668 ± 0.06	0.650 ± 0.04	0.657 ± 0.08	0.638 ± 0.06
4	Regression-FR	—	0.686 ± 0.02	—	0.644 ± 0.02	—	0.618 ± 0.03	—	0.566 ± 0.05
5	Regression-FN	—	0.714 ± 0.02	—	0.675 ± 0.04	—	0.636 ± 0.03	—	0.592 ± 0.05

TABLE VII

SCAN-LEVEL PERFORMANCE (MEAN ± STD.DEV) FOR ABLATION ON THE ENCODER-DECODER ARCHITECTURE. EACH NETWORK IS PRE-TRAINED WITH MORPH-SSL AND EVALUATED BY EITHER FREEZING (FR) WEIGHTS OR END-TO-END FINETUNING (FN) ON THE DOWNSTREAM TASK. THE BEST VALUE IN EACH COLUMN IS HIGHLIGHTED IN BOLD. THE STATISTICAL SIGNIFICANCE OF THE AUC VALUES IN ROWS 2-4 IS COMPARED WITH ROW 1 AND ROWS 6-8 WITH ROW 5 WITH THE DELONG TEST. THE VALUES HIGHLIGHTED WITH * ARE **NOT** STATISTICALLY DIFFERENT WITH $p > 0.05$

SL.		0 month		6 month		12 month		18 month	
No.		AUC	Bal Acc.	AUC	Bal Acc.	AUC	Bal Acc.	AUC	Bal Acc.
1	Proposed-FR	$\mathbf{0.876 \pm 0.02}$	0.810 ± 0.02	0.767 ± 0.02	0.721 ± 0.01	0.718 ± 0.04	0.686 ± 0.02	0.693 ± 0.06	0.673 ± 0.04
2	3D Convolutions-FR	0.859 ± 0.03	0.801 ± 0.02	0.744 ± 0.02	0.695 ± 0.01	0.695 ± 0.04	0.663 ± 0.03	0.679 ± 0.06	0.653 ± 0.04
3	BatchNorm-FR	0.850 ± 0.03	0.803 ± 0.02	0.743 ± 0.02	0.705 ± 0.02	0.699 ± 0.04	0.668 ± 0.03	$0.690 \pm 0.06^*$	0.667 ± 0.04
4	Additive skip conn-FR	0.845 ± 0.03	0.789 ± 0.03	0.743 ± 0.02	0.694 ± 0.02	0.686 ± 0.03	0.657 ± 0.02	0.661 ± 0.04	0.643 ± 0.03
5	Proposed-FN	0.871 ± 0.02	$\mathbf{0.812 \pm 0.02}$	$\mathbf{0.768 \pm 0.02}$	$\mathbf{0.727 \pm 0.01}$	$\mathbf{0.721 \pm 0.05}$	$\mathbf{0.691 \pm 0.03}$	0.700 ± 0.06	$\mathbf{0.674 \pm 0.05}$
6	3D Convolutions-FN	0.858 ± 0.02	0.795 ± 0.03	0.748 ± 0.01	0.706 ± 0.02	0.698 ± 0.04	0.671 ± 0.02	0.684 ± 0.06	0.663 ± 0.04
7	BatchNorm-FN	0.846 ± 0.03	0.793 ± 0.02	0.750 ± 0.02	0.704 ± 0.02	0.710 ± 0.03	0.680 ± 0.01	$\mathbf{0.701 \pm 0.04}^*$	0.673 ± 0.01
8	Additive skip conn-FN	0.841 ± 0.03	0.777 ± 0.02	0.740 ± 0.02	0.697 ± 0.01	0.687 ± 0.04	0.658 ± 0.02	0.653 ± 0.05	0.639 ± 0.04

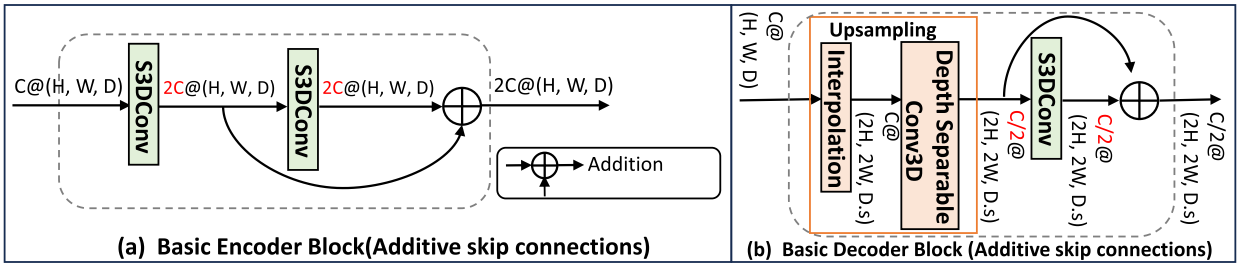

Fig. 7. The alternative architectures with additive skip connections used to replace the proposed Basic Encoder and Decoder Blocks (see Fig. 2(b), (d)) in the ablation experiments presented in Table II, VII.

TABLE VIII

SCAN-LEVEL PERFORMANCE (MEAN ± STD. DEVIATION) FOR ABLATION ON THE DOWNSTREAM TTC CLASSIFICATION LOSS (ROWS 2-3) AND THE CLASSIFIER ARCHITECTURE (ROWS 4-5). THE MORPH-SSL PRE-TRAINED ENCODER WEIGHTS ARE FROZEN AND ONLY THE CLASSIFIER IS TRAINED IN EACH EXPERIMENT. THE BEST VALUE IN EACH COLUMN IS HIGHLIGHTED IN BOLD. THE AUC VALUES IN ROWS 2-5 ARE COMPARED WITH ROW 1 USING THE DELONG TEST AND THE VALUES WHICH ARE **NOT** STATISTICALLY SIGNIFICANT ($p > 0.05$) ARE HIGHLIGHTED WITH *

SL.		0 month		6 month		12 month		18 month	
No.		AUC	Bal Acc.	AUC	Bal Acc.	AUC	Bal Acc.	AUC	Bal Acc.
1	Proposed	0.876 ± 0.02	0.810 ± 0.02	$\mathbf{0.767 \pm 0.02}$	$\mathbf{0.721 \pm 0.01}$	$\mathbf{0.718 \pm 0.04}$	$\mathbf{0.686 \pm 0.02}$	$\mathbf{0.693 \pm 0.06}$	$\mathbf{0.673 \pm 0.04}$
2	no $\|\|a\|\|_2^2$	$0.869 \pm 0.02^*$	0.809 ± 0.02	0.753 ± 0.02	0.706 ± 0.01	0.705 ± 0.04	0.666 ± 0.02	0.679 ± 0.06	0.653 ± 0.04
3	no $\|\|\mathbf{M} \odot (1 - \widehat{\mathbf{R}})\|\|_1$	0.865 ± 0.03	0.808 ± 0.02	0.755 ± 0.02	0.711 ± 0.01	$0.714 \pm 0.05^*$	0.685 ± 0.02	$0.692 \pm 0.06^*$	0.671 ± 0.03
4	Multilabel Classifier	$\mathbf{0.885 \pm 0.01}$	$\mathbf{0.841 \pm 0.01}$	$0.763 \pm 0.02^*$	0.720 ± 0.02	0.709 ± 0.05	0.681 ± 0.02	0.686 ± 0.06	0.667 ± 0.03
5	Separate a prediction	$0.869 \pm 0.03^*$	0.812 ± 0.03	$0.766 \pm 0.02^*$	0.715 ± 0.02	$0.717 \pm 0.04^*$	0.684 ± 0.02	$0.689 \pm 0.05^*$	0.659 ± 0.03

TABLE IX

SCAN-LEVEL PERFORMANCE (MEAN±STD. DEVIATION) TO BENCHMARK THE PROPOSED METHOD (FINE-TUNED FROM MORPH-SSL PRE-TRAINED WEIGHTS) AGAINST STANDARD 3D NETWORKS (FINE-TUNED FROM THEIR AVAILABLE WEIGHTS PRE-TRAINED ON THE KINETICS DATASET) AND HANDCRAFTED BIOMARKERS WITH A RANDOM FOREST CLASSIFIER. THE BEST PERFORMANCE IN EACH COLUMN IS HIGHLIGHTED IN BOLD. THE AUC DIFFERENCE OF ROWS 2-4 COMPARED TO ROW 1 WAS FOUND TO BE STATISTICALLY SIGNIFICANT ($p < 0.05$) FOR ALL TIME-POINTS USING THE DELONG TEST

SL. No.		0 month AUC	0 month Bal Acc.	6 month AUC	6 month Bal Acc.	12 month AUC	12 month Bal Acc.	18 month AUC	18 month Bal Acc.
1	Proposed-Finetune	$\mathbf{0.871 \pm 0.02}$	$\mathbf{0.812 \pm 0.02}$	$\mathbf{0.768 \pm 0.02}$	$\mathbf{0.727 \pm 0.01}$	$\mathbf{0.721 \pm 0.05}$	$\mathbf{0.691 \pm 0.03}$	$\mathbf{0.700 \pm 0.06}$	$\mathbf{0.674 \pm 0.05}$
2	I3D [45]	0.796 ± 0.05	0.752 ± 0.04	0.701 ± 0.02	0.677 ± 0.01	0.657 ± 0.03	0.640 ± 0.01	0.654 ± 0.03	0.625 ± 0.02
3	X3D [46]	0.792 ± 0.01	0.756 ± 0.02	0.710 ± 0.01	0.683 ± 0.01	0.673 ± 0.02	0.650 ± 0.02	0.666 ± 0.02	0.645 ± 0.01
4	Biomarkers+Random Forest	0.753 ± 0.03	0.708 ± 0.02	0.673 ± 0.04	0.653 ± 0.04	0.628 ± 0.05	0.619 ± 0.04	0.609 ± 0.06	0.607 ± 0.04

TABLE X

SCAN-LEVEL AREA UNDER THE ROC CURVE (MEAN±STD. DEVIATION) TO COMPARE SSL METHODS UNDER DIFFERENT TRAINING CONFIGURATIONS BY: EITHER TRAINING ON ONE-THIRD OR THE ENTIRE TRAINING DATASET; EITHER FREEZING SSL-TRAINED WEIGHTS OR FINETUNING END-TO-END. THE VALUES HIGHLIGHTED WITH * IN EACH COLUMN ARE **NOT** STATISTICALLY DIFFERENT ($p > 0.05$) COMPARED TO THE PROPOSED METHOD TRAINED WITH IDENTICAL DATA (EITHER ONE-THIRD OR THE ENTIRE DATASET) AND PROTOCOL (EITHER FREEZE SSL WEIGHTS OR FINETUNE). THE BEST PERFORMANCE IN EACH COLUMN IS HIGHLIGHTED IN **BOLD**

	One-third Training data				Entire Training data			
	0 month	6 month	12 month	18 month	0 month	6 month	12 month	18 month
Proposed-Freeze	0.842 ± 0.04	0.745 ± 0.03	0.697 ± 0.05	0.679 ± 0.06	$\mathbf{0.876 \pm 0.02}$	0.767 ± 0.02	0.718 ± 0.04	0.693 ± 0.06
Proposed-Finetune	$\mathbf{0.864 \pm 0.02}$	$\mathbf{0.764 \pm 0.03}$	$\mathbf{0.715 \pm 0.05}$	$\mathbf{0.690 \pm 0.06}$	0.871 ± 0.02	$\mathbf{0.768 \pm 0.02}$	$\mathbf{0.721 \pm 0.05}$	$\mathbf{0.700 \pm 0.06}$
Model Genesis-Freeze [3]	0.801 ± 0.02	0.704 ± 0.01	0.659 ± 0.02	0.648 ± 0.03	0.820 ± 0.02	0.717 ± 0.01	0.670 ± 0.03	0.658 ± 0.05
Model Genesis-Finetune [3]	0.783 ± 0.03	0.698 ± 0.04	0.653 ± 0.06	0.626 ± 0.06	0.846 ± 0.02	0.744 ± 0.02	$\mathbf{0.721 \pm 0.04}^*$	$0.699 \pm 0.06^*$
Time prediction-Freeze [14]	0.600 ± 0.04	0.542 ± 0.04	0.521 ± 0.06	0.522 ± 0.05	0.715 ± 0.06	0.590 ± 0.07	0.540 ± 0.07	0.536 ± 0.07
Time prediction-Finetune [14]	0.646 ± 0.12	0.610 ± 0.09	0.580 ± 0.07	0.565 ± 0.06	0.759 ± 0.05	0.655 ± 0.03	0.615 ± 0.02	0.583 ± 0.03
Barlow Twins-Freeze [10]	0.776 ± 0.03	0.669 ± 0.03	0.598 ± 0.03	0.559 ± 0.06	0.751 ± 0.03	0.678 ± 0.03	0.635 ± 0.03	0.612 ± 0.04
Barlow Twins-Finetune [10]	0.775 ± 0.05	0.670 ± 0.04	0.628 ± 0.05	0.610 ± 0.07	0.774 ± 0.03	0.679 ± 0.04	0.641 ± 0.04	0.614 ± 0.04
VICReg-Freeze [9]	0.783 ± 0.04	0.702 ± 0.02	0.668 ± 0.03	0.658 ± 0.03	0.842 ± 0.02	0.733 ± 0.01	0.684 ± 0.02	0.659 ± 0.03
VICReg-Finetune [9]	0.822 ± 0.03	0.738 ± 0.03	0.693 ± 0.04	$0.684 \pm 0.05^*$	0.852 ± 0.02	0.755 ± 0.01	0.700 ± 0.02	0.680 ± 0.03

A. Limitations and Future Directions

The large amount of time required for training multiple 3D CNN networks prevented an exhaustive search for the optimal network architecture, the size of Encoder's output feature map, and the tunable weights of the loss terms used during Morph-SSL training, which remains a limitation of this work. Currently, the classification network for forecasting the conversion risk treated each scan acquired at different time-points of the same eye as independent training samples. Although the current model showed a moderate amount of consistency between predictions from different visits for the same future time-point (eCI = 0.73), an alternate approach for the supervised downstream task training may be explored in the future to explicitly enforce this consistency constraint. Finally, although Morph-SSL was primarily developed to pre-train the Encoder in an unsupervised manner, the learned Encoder-Decoder network can additionally smoothly interpolate between the scans from two visits. This offers promising future research directions for using the interpolated scans as a data augmentation or to visualize the expected future morphological changes if a Recurrent Neural Network could be trained to predict the feature representations of future visits. Adapting Morph-SSL to other prognostic tasks in the medical domain, such as forecasting cancer progression from ultrasound images or predicting the future onset of dementia from MRI scans, offers important directions for future work.

APPENDIX

See Tables VI–X and Fig. 7.
